# Photocatalytically active ladder polymers†
†Electronic supplementary information (ESI) available. See DOI: 10.1039/c8fd00197a


**DOI:** 10.1039/c8fd00197a

**Published:** 2019-01-22

**Authors:** Anastasia Vogel, Mark Forster, Liam Wilbraham, Charlotte L. Smith, Alexander J. Cowan, Martijn A. Zwijnenburg, Reiner Sebastian Sprick, Andrew I. Cooper

**Affiliations:** a Department of Chemistry , Materials Innovation Factory , University of Liverpool , Liverpool , UK . Email: aicooper@liverpool.ac.uk; b Department of Chemistry , Stephenson Institute for Renewable Energy , University of Liverpool , Liverpool , UK; c Department of Chemistry , University College London , London , UK

## Abstract

Conjugated ladder polymers (cLaPs) are introduced as organic semiconductors for photocatalytic hydrogen evolution from water under sacrificial conditions. Starting from a linear conjugated polymer (cLiP1), two ladder polymers are synthesized *via* post-polymerization annulation and oxidation techniques to generate rigidified, planarized materials bearing dibenzo[*b*,*d*]thiophene (cLaP1) and dibenzo[*b*,*d*]thiophene sulfone subunits (cLaP2). The high photocatalytic activity of cLaP1 (1307 μmol h^–1^ g^–1^) in comparison to that of cLaP2 (18 μmol h^–1^ g^–1^) under broadband illumination (*λ* > 295 nm) in the presence of a hole-scavenger is attributed to a higher yield of long-lived charges (μs to ms timescale), as evidenced by transient absorption spectroscopy. Additionally, cLaP1 has a larger overpotential for proton reduction and thus an increased driving force for the evolution of hydrogen under sacrificial conditions.

## Abstract 2

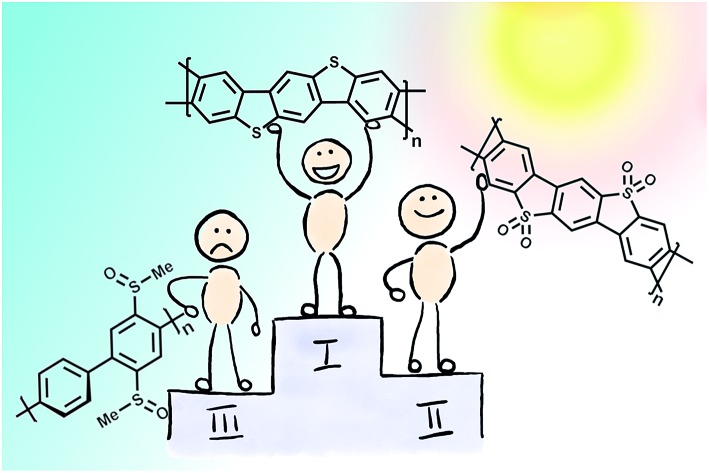
Post-polymerization ladderization is explored as a promising technique to boost the photo-catalytic activity of conjugated polymers.

## Introduction

Clean and sustainable production of hydrogen is one promising strategy for future zero-emission energy supply.^1^ In this context, photocatalysis using heterogeneous semiconductors for water splitting has received much attention. Progress has been made in the application of both inorganic^2^ and organic semiconductors, the latter triggered by studies on carbon nitride,^3^ which have inspired many follow-up studies.^4^ The majority of studies focus on half reactions using sacrificial agents to produce either hydrogen or oxygen, but overall water-splitting systems have also been reported that produce both gases.^5^ Conjugated polymer semiconductors have gained much attention recently^6^ because of their synthetic modularity, the large number of monomers that are available, and the resulting tunability in their physical properties. This has triggered the development of a plethora of new types of polymer photocatalysts, including conjugated linear polymers (cLiPs)‡
‡In the literature, both conjugated linear and conjugated ladder polymers are abbreviated as cLPs.^20^ For the purposes of this paper, we will use the abbreviation cLiP for linear polymers and cLaP for ladderized polymers.,^7^,^8^ conjugated microporous polymers (CMPs),^9^–^11^ conjugated triazine frameworks (CTFs)^12^ and covalent organic frameworks (COFs).^13^,^14^ The modularity of these materials over a wide range of monomer building blocks allows the transfer of photocatalytically active subunits from one class of materials into another. This allows us, in principle, to build structure–property relationships where molecular effects are deconvoluted from solid state packing effects. A complication is that the efficacy of heterogeneous polymer photocatalysts depends on a large number of independent variables, including but not limited to the extent of conjugation and the light absorption cross-section,^15^ the residual metal content,^9^,^11^ wettability,^16^ thermodynamic driving forces for proton reduction,^15^,^17^ and charge carrier life-times.^18^ However, none of these variables have so far been singled out as the most dominant one: instead photocatalytic activity is a complex function of many different interrelated factors, often thwarting attempts to design better catalysts.

**Fig. 1:**
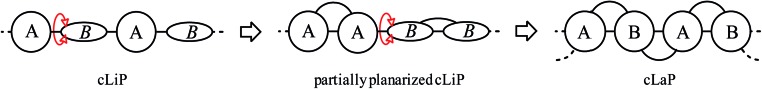
Graphical representation of conjugated linear polymer (cLiP) with free torsional motions, partially planarized cLiP, and conjugated ladder polymer (cLaP).

**Fig. 2:**
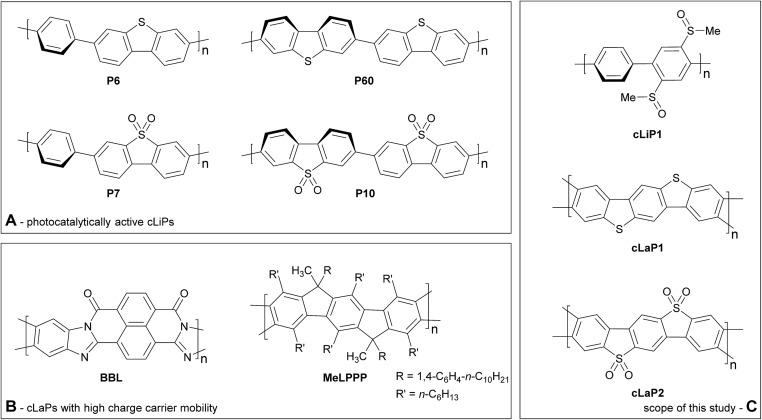
(a) Photocatalytically active conjugated linear polymers containing dibenzo[*b*,*d*]thiophene and dibenzo[*b*,*d*]thiophene sulfone building blocks; (b) conjugated ladder polymers with reported high charge carrier mobilities; (c) scope of this study including parent linear conjugated polymer **cLiP1** and conjugated ladder polymers **cLaP1** and **cLaP2**.

This does not mean that there are no viable structural hypotheses for polymer photocatalyst design. For example, previous studies on fluorene-type polymers suggest that partial planarization of poly(*p*-phenylene) leads to an increase in photocatalytic activity (Fig. 1).^8^ Conjugated linear polymers can be further planarized forming a double-stranded polymer; that is, a so-called ‘conjugated ladder polymer’ (cLaP)^19^–^21^ Ladder polymers restrict the free torsional motion between the monomer units and, in the case of cLaPs (Fig. 1; also **BBL** and **MeLPPP**, Fig. 2B), this leads to a fully coplanar, π-conjugated polymer backbone.^20^ Thus, cLaPs tend to exhibit high thermal, optical, and mechanical stability, as well as high resistance to chemical degradation and π-conjugation, along with long exciton diffusion lengths and strong π–π stacking interactions.^19^,^20^ In principle, all of these features are desirable in a polymer photocatalyst. Ladder polymers such as **BBL** (Fig. 2B) have been shown to have high electron mobilities when compared to the conjugated, non-ladderized parent polymer **BBB**.^22^ Also, the degree of order within the conjugated ladder polymer **MeLPPP** has been highlighted to be a major contributor to its high charge carrier mobility, which resembles molecular crystals more than conventional, less ordered conjugated polymers.^23^

Conjugated linear polymers containing dibenzo[*b*,*d*]thiophene sulfone units (as in **P7**, Fig. 2A) have been shown repeatedly to outperform most other organic photocatalysts.^8^,^14^,^24^–^26^ This is attributed to the co-planarization of neighbouring subunits in the polymer, increased hydrophilicity, and strong visible light absorption.^8^,^14^ While there is one report on the use of **BBL** as a photoanode for photoelectrochemical water splitting,^27^ no ladder polymers have been reported as bulk powdered photocatalysts for direct water splitting.

Here, we set out to combine structural features derived from highly active linear polymer photocatalysts with the increased conjugation that might be expected for a conjugated ladder polymer. We did this by synthesizing and analyzing a series of related linear (**cLiP1**) and ladder polymers based on dibenzo[*b*,*d*]thiophene (**cLaP1**) and dibenzo[*b*,*d*]thiophene sulfone (**cLaP2**) units (Fig. 2C), followed by testing these materials for sacrificial photocatalytic water reduction.

## Results

### Synthesis and characterization

**Scheme 1:**
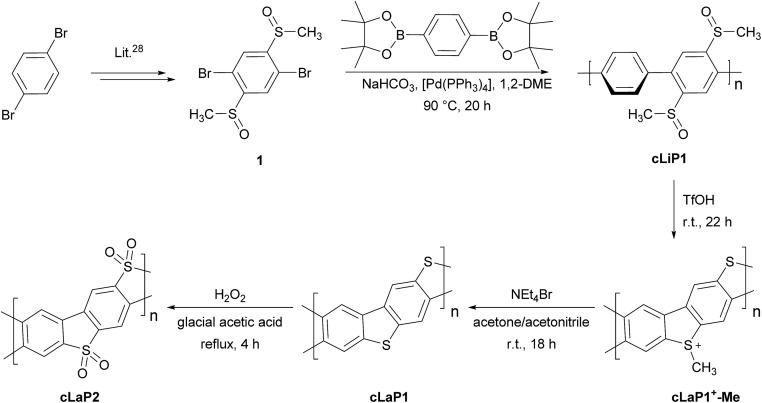
The synthesis route of conjugated linear polymer **cLiP1** and the related conjugated ladder polymers **cLaP1** and **cLaP2**.

In contrast to the cross-coupling reactions (*e.g.*, Suzuki–Miyaura, Stille or Kumada coupling) that are typically used to yield linear conjugated polymers in one step, ladder polymers are usually synthesized by either (A) a single-step polycondensation reaction of tetra-functionalized building blocks or (B) polymerization and subsequent annulation of a bi-functionalized subunit to give a ladderized polymer.^20^ For the synthesis of ladder polymers containing dibenzo[*b*,*d*]thiophene and dibenzo[*b*,*d*]thiophene sulfone, polymerization of an aryl bi-sulfoxide **1***via* route B was chosen. Building block **1** was synthesized from 1,4-dibromobenzene (Scheme 1) according to the literature.^28^ Polymerization of **1** with 1,4-benzene diboronic acid ester *via* a Pd(0)-catalyzed Suzuki–Miyaura cross-coupling reaction gave the parent polymer **cLiP1**.^29^ An intramolecular ring-closing reaction was then performed using trifluoromethanesulfonic acid (TfOH),^29^ and the intermediate polysulfonium salt (**cLaP^+^-Me**) was dealkylated using NEt_4_Br to give the ladderized polymer **cLaP1**. Further oxidation of **cLaP1** with H_2_O_2_ in glacial acetic acid gives **cLaP2**, which is a ladder-type analogue of the linear dibenzo[*b*,*d*]thiophene sulfone polymer (**P10**).^30^^1^H{^13^C} NMR and IR spectra for previously reported compounds (see the Experimental section of the ESI and Fig. S10 and S11†) agree with the literature. It is worth noting that no convenient method is available to quantify the degree of ladderization and degree of dealkylation since all materials were insoluble in common organic solvents. The total insolubility of these materials also precludes molecular weight determination.

Fourier-transform infrared spectroscopy (FT-IR) was used to analyze the insoluble polymers: **cLiP1** has a stretching vibration at 1032 cm^–1^ that can be attributed to the sulfoxide group (*ν̃*_S=O_) and, in the fingerprint region, sharp peaks (837, 747, 674 cm^–1^) corresponding to wagging C–H and C–C vibrations for 1,4- and 1,2,4,5-substituted benzene subunits are observed (Fig. S10†). Upon ladderization to **cLaP1**, the spectrum is dominated by strong peaks attributed to various stretching vibrations of the triflate anion (635, 1224 cm^–1^ (*ν̃*_C–F_) and 1156 cm^–1^ (*ν̃*_O=S=O_), see also Fig. S11†). The fingerprint region shows a single broad peak at 832 cm^–1^ consistent with a polymer containing 1,2,4,5-substituted benzene subunits. The signals of the triflate anion (compare Fig. S11,† right) indicate that not all of the intermediate sulfonium subunits were demethylated when treated with NEt_4_Br, despite further attempts to optimize the conditions. Furthermore, the presence or absence of the sulfoxide group (1010 cm^–1^ (*ν̃*_S=O_)) after the ladderization could not be detected due to the overlapping signals of the triflate anion. Thus, no further conclusions on the success rate of the annulation or the presence of defects (non-annulated aryl sulfoxide groups) could be made at this point. Upon oxidation to **cLaP2**, the spectrum shows two strong stretching vibrations (1307 cm^–1^ and 1148 cm^–1^; symmetric and asymmetric *ν̃*_O=S=O_) indicating the oxidation of the dibenzo[*b*,*d*]thiophene moiety to dibenzo[*b*,*d*]thiophene dioxide (Fig. S12†). Additionally, no signals associated with the presence of triflate anions were observed. There is also no evidence for over-oxidation nor ring-opening reactions of the polymer to yield sulfonic acid groups.

**Fig. 3:**
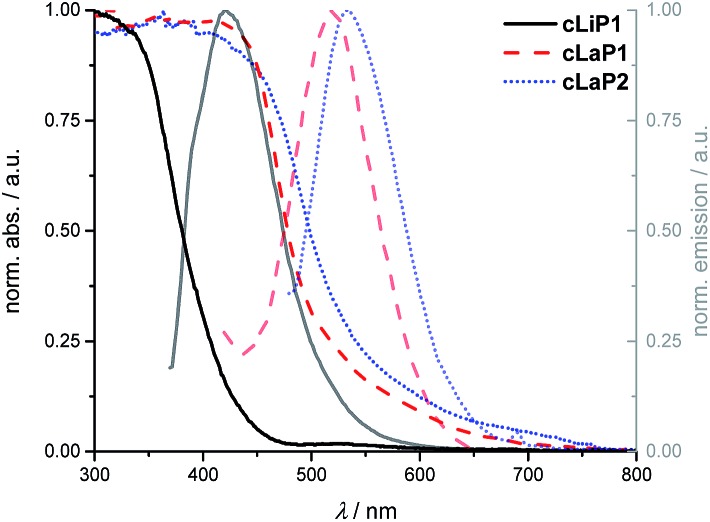
Normalized UV-vis absorption and photoluminescence emission spectra of **cLiP1** (black, full lines), **cLaP1** (red, dashed lines) and **cLaP2** (blue, dotted lines) from powder samples.

Powder X-ray diffraction patterns (PXRD) show that polymer **cLiP1** has limited long-range order, while **cLaP1** and **cLaP2** are amorphous (Fig. S17–S19†). Scanning electron microscopy (SEM) shows that all of the materials consist of *ca*. 100 nm spheres that are fused together (Fig. S22†). UV-vis and photoluminescence (PL) spectroscopy were used to probe the optoelectronic properties of **cLiP1**, **cLaP1** and **cLaP2** measured as powders in the solid-state (Fig. 3 and Fig. S1–S9†): polymer **cLiP1** absorbs mostly in the UV region with an absorption edge around 415 nm. Upon ladderization of **cLiP1**, the absorption edge shifts by about 100 nm for **cLaP1**§
§UV-vis and fluorescence spectra for **cLaP1** were recorded using material recovered from experiments for hydrogen evolution as this represents the active catalysts best. A UV-vis spectrum of the as-synthesized **cLaP1** (or rather **cLaP1^+^-Me**) can be found in the ESI.†
 and this clearly shows that the system has a higher degree of conjugation as the annulation of neighbouring conjugated units reduces their respective torsion angles close to zero degrees as the system is rigidified (compare Fig. S39†). As expected,^8^ the oxidation to **cLaP2** led to only minor changes in the UV-vis spectrum. Polymer **cLiP1** has an estimated optical gap of 3.01 eV, while both ladderized polymers **cLaP1** and **cLaP2** show narrower optical gaps of 2.41 and 2.30 eV (for Tauc plots see Fig. S1–S5†), respectively.

The fluorescence emission spectra in the solid state show the same bathochromic shifts as the UV-vis absorption spectra. Moreover, a smaller Stokes’ shift and resolved vibronic coupling in the excitation spectrum of **cLaP2** and **cLaP1** compared to **cLiP1** (Fig. S6–S9†) underlines once more the increased rigidity (and symmetry) of the polymer upon ladderization. Time-correlated single photon counting (TCSPC) was used to estimate the lifetime of the excited state in an aqueous suspension (Fig. S32–S34 and Table S3†). Polymer **cLiP1** has a short lifetime of 0.14 ns, which is similar to that of **cLaP1** with 0.21 ns. The dibenzo[*b*,*d*]thiophene sulfone polymer **cLaP2** has the longest lifetime of the materials studied (*τ*_avg_ = 1.71 ns), as observed previously.^31^

### Photocatalytic performance

**Fig. 4:**
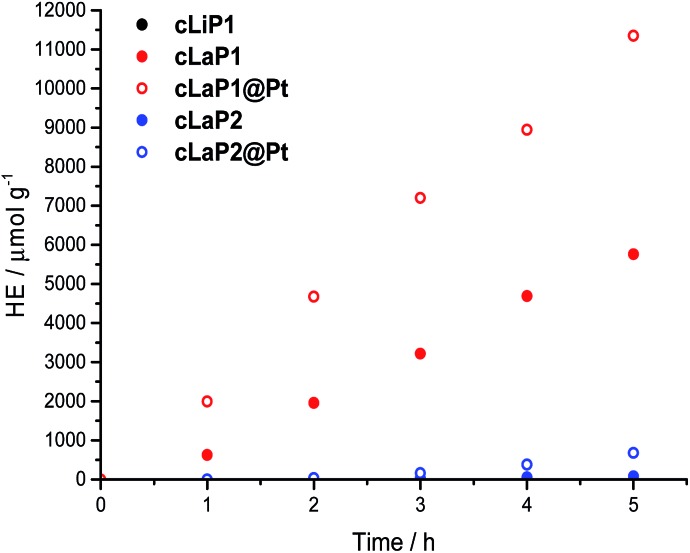
Hydrogen evolution of **cLiP1**, **cLaP1 **and **cLaP2** as well as **cLaP1@Pt** and **cLaP2@Pt** from a H_2_O/TEA/MeOH mixture under broadband irradiation (300 W Xe light source, *λ* > 295 nm).

The photocatalytic activity of these materials for hydrogen evolution from water in the presence of triethylamine (TEA) as a sacrificial electron donor was studied under broad-spectrum and visible light irradiation (*λ* > 295 nm and *λ* > 420 nm; Fig. 4 and Table 1). In addition, methanol was used in the aqueous mixture to enhance miscibility of TEA with water, and to improve wettability of the polymers.^8^,^33^ Polymer **cLiP1** showed very limited activity (15 μmol h^–1^ g^–1^), even compared to poly(*p*-phenylene) (232 μmol h^–1^ g^–1^).^8^ Upon ladderization to **cLaP1**, the photocatalytic activity increased dramatically to 1307 μmol h^–1^ g^–1^. When the material was recovered and used again as a photocatalyst, an increase in the hydrogen evolution rate to over 2000 μmol h^–1^ g^–1^ was observed (Fig. S32†). This can possibly be explained by further demethylation of the catalyst during catalysis by TEA, which reduces the doping levels of the photocatalyst, and a lower effective mass for the recovered catalyst and thus higher HER per gram. This is supported by post-catalysis FT-IR measurements (Fig. S14†): peaks associated with the presence of the triflate counterion are no longer present, and thus a no longer charged polymer species has to be assumed after irradiation. The oxidation of **cLaP1** to **cLaP2**, led to an almost total loss of activity (18 μmol h^–1^ g^–1^).

**Table 1:** Photophysical properties, hydrogen evolution rates and palladium content

Polymer	*λ*_edge_ ^*a*^/nm	*E* Tauc g ^*b*^/eV	*λ*_em_ ^*c*^/nm	HER (*λ* > 295 nm) ^*d*^/μmol h^–1^ g^–1^	HER (*λ* > 420 nm) ^*d*^/μmol h^–1^ g^–1^	Pd content ^*e*^/wt%
**cLiP1**	415	3.01	433	15 ± 2	0^*f*^	0.83
**cLaP1**	514	2.41	520	1307 ± 26	317 ± 9	0.38
**cLaP1@Pt**	—	—	—	2297 ± 92	1489 ± 24	0.38 (+1 Pt)^*g*^
**cLaP2**	548	2.30	534	18 ± 1	0^*f*^	0.36
**cLaP2@Pt**	—	—	—	272 ± 10	184 ± 7	0.36 (+1 Pt)^*g*^
**P60**	454	2.68	460	1295 ± 36	641 ± 20	0.49
**P60@Pt**	—	—	—	1703 ± 102	213 ± 5	0.49 (+1 Pt)^*g*^
**P6** (Lit. 8)	448	—	456, 481	1660 ± 12	432 ± 4	0.60
**P7** (Lit. 8)	459	—	477	2352 ± 76	1492 ± 32	0.38
**P10** (Lit. 26)	473	—	509	—	3260 ± 164	0.40

^*a*^Absorption onset determined from UV-vis reflectance measurements in the solid state.

^*b*^Optical gap determined from absorption spectra using the Tauc method.^32^

^*c*^Emission peak maximum determined in the solid state.

^*d*^Hydrogen evolution rate determined in H_2_O/TEA/MeOH irradiated with a 300 W Xe light source using suitable filters.

^*e*^Palladium content determined *via* ICP-MS.

^*f*^No hydrogen was detected with five hours of irradiation.

^*g*^Platinum was photodeposited *in situ* onto the polymer from H_2_PtCl_6_.

All of the materials were tested as synthesized without the addition of any additional metal co-catalyst. However, it has been shown that residual palladium originating from the Suzuki–Miyaura coupling reaction can act as a co-catalyst.^9^,^34^ Inductively coupled plasma mass spectrometry (ICP-MS) measurements show that the residual palladium content decreased from 0.83 wt% for **cLiP1** to 0.38 and 0.36 wt% for **cLaP1** and **cLaP2**, respectively. The lower residual palladium amount might be due to the use of trifluoromethanesulfonic acid and repeated washing of **cLaP1** after the ring-closure reaction. Similarly, the oxidation giving **cLaP2** is performed in acetic acid. The use of acids has been previously shown to decrease the residual palladium loadings of insoluble conjugated microporous polymers.^35^ Finally, the photocatalytic activity of **cLaP1** was increased from 317 to 1489 μmol h^–1^ g^–1^ under visible light illumination and from 1307 to 2297 μmol h^–1^ g^–1^ under broadband illumination by *in situ* photodeposition of platinum as co-catalyst (**cLaP1@Pt**, 1 wt%). Similarly, **cLaP2** showed a 10-fold increase in activity (from 18 to 272 μmol h^–1^ g^–1^) under broadband illumination with platinum as co-catalyst (**cLaP2@Pt**, 1 wt%).

External quantum efficiencies (EQEs) were estimated for **cLaP1** in H_2_O/TEA/MeOH mixtures using monochromatic light and these show that the hydrogen production is indeed photocatalytic (Fig. S31†). At 420 nm an EQE of 1.6% was determined, which increased to 2.8% upon addition of platinum (**cLaP1@Pt**, 1 wt%). This is higher than previously reported for **P1** (EQE_420 nm_ = 0.4%)^26^ and biphenyl-thiophene-co-polymer **P12** (EQE_420 nm_ = 1.4%)^15^ under the same conditions, but lower than that of phenylene-benzothiadiazole-co-polymer **B-BT-1,4** (EQE_420 nm_ = 4.0%)7*a* in triethanolamine/water mixture loaded with platinum, and phenylene–dibenzo[*b*,*d*]thiophene sulfone **P7** (EQE_420 nm_ = 7.2%)^26^ in a H_2_O/TEA/MeOH mixture.

### Transient absorption spectroscopy

**Fig. 5:**
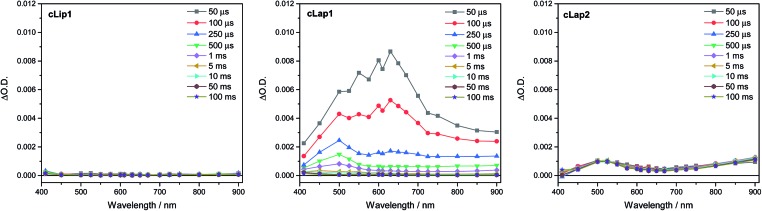
μs to ms TA spectra (same scale on all *y*-axes) of **cLiP1**, **cLaP1** and **cLaP2** suspended in a H_2_O/TEA/MeOH mixture following excitation with a 355 nm laser (6 ns, 400 μJ cm^–2^, 0.33 Hz). The large TA features seen with **cLaP1** correlate with the high rate of H_2_ evolution.

**Fig. 6:**
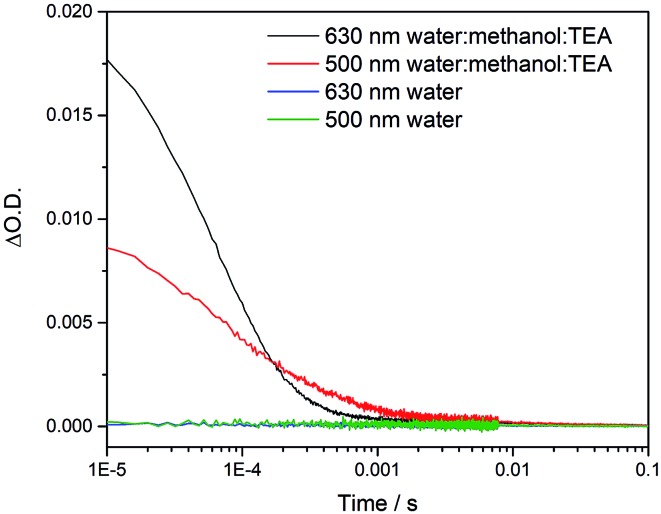
Kinetic traces recorded at the wavelengths indicated for **cLaP1** in either water alone or a H_2_O/TEA/MeOH mixture under a nitrogen atmosphere following excitation with a 355 nm (6 ns, 400 μJ cm^–2^, 0.33 Hz) laser.

To try to explain the differences in catalytic activity, we studied the charge carrier lifetimes using transient absorption (TA) spectroscopy. TA has been shown to be an effective tool for studying the formation and lifetime of electron–polaron states in polymer photocatalysts for hydrogen evolution.^26^,^36^ Here we focused on the kinetics of species present on the μs to ms timescales following UV (355 nm) excitation of **cLiP1**, **cLaP1** and **cLaP2** in the presence of a H_2_O/TEA/MeOH mixture under a nitrogen atmosphere (Fig. 5). All three materials exhibited transient absorptions between 400–900 nm on the timescale probed, however the amplitude of the TA signal of **cLaP1** was far greater (×5–10) than those of **cLiP1** and **cLaP2**, indicating an increase in long-lived photogenerated species. The TA spectrum of **cLaP1** contains two distinct absorptions centred at *ca.* 500 and 630 nm that decay at different rates (Fig. S35 and S36,† *t*_50%_ ∼ 18 μs (500 nm), 25 μs (630 nm) under N_2_).¶
¶We note that the lifetimes of all of the bands are dependent upon the history of the sample and tend to decrease after prolonged experiments. However, the signal at 500 nm is consistently shorter-lived compared to the signal at 625 nm. In the absence of methanol and TEA the long-lived TA bands at 500 and 630 nm are no longer observed (Fig. 6). The role of TEA has previously been investigated with ultrafast TA spectroscopy^26^,^36^ and it was shown to be required for efficient hole scavenging and for the formation of long-lived electron polarons that are suitable for proton reduction. In the absence of TEA, the excitonic states would be expected to decay rapidly after formation on timescales faster than those studied here (ps to ns), in line with TCSPC measurements.^37^ We therefore propose that the bands at 500 and 630 nm are due to two distinct electron populations within the sample **cLaP1**. Supporting our assignment are TA experiments carried out using oxygen as an electron scavenger. Introduction of O_2_ into the system significantly decreases the TA signal at both 500 and 630 nm indicating the removal of electron populations (Fig. S37†). The observation of a long-lived electron signal at 630 nm for **cLaP1** is in line with a recent assignment of an electron polaron state in related dibenzo[*b*,*d*]thiophene sulfone linear polymers.^26^ However, the assignment of both individual TA features to specific electron populations, potentially related to the presence of some residual sulfonium subunits, is challenging as the transient UV/vis spectra measured contain broad bands and our experimental resolution prevents the observation of fine structure.

Although the magnitudes of the TA signals of **cLaP2** are far smaller than those of **cLaP1** on the μs timescale, the kinetic traces recorded at 500 nm for **cLaP2** do indicate an extremely long-lived (*t*_50%_ = 0.3 s) photogenerated species. The presence of extremely long-lived charges, potentially with a low thermodynamic driving force, may also be a factor behind the low HER observed for **cLaP2**. To explore this observation further, Pt was added as a co-catalyst in the hope that it may be able to either intercept photoelectrons and prevent trapping or offer suitable catalytic sites to facilitate charge transfer into solution. **cLaP2@Pt** does show an improvement in HER, increasing from 18 μmol h^–1^ g^–1^ to 272 μmol h^–1^ g^–1^ (Table 1), but a comparison of the TA spectra of **cLaP2** and **cLaP2@Pt** shows no clear difference under nitrogen (Fig. S38†), suggesting that the charges observed at 500 nm do not transfer to Pt and that the hydrogen evolution catalysed with Pt happens either on a sub 10 μs timescale or that the electron population has absorption features outside of our spectral window. The addition of a Pt co-catalyst also increases the rate of hydrogen evolution in **cLap1** (Table 1). In this case, the increase in HER evolution following Pt addition is accompanied by a significant decrease in the TA magnitudes at both 500 and 630 nm (Fig. S37†) indicating that these previously long-lived charges are now transferring to Pt and on timescales faster than those in our experiment.

### Calculations

**Fig. 7:**
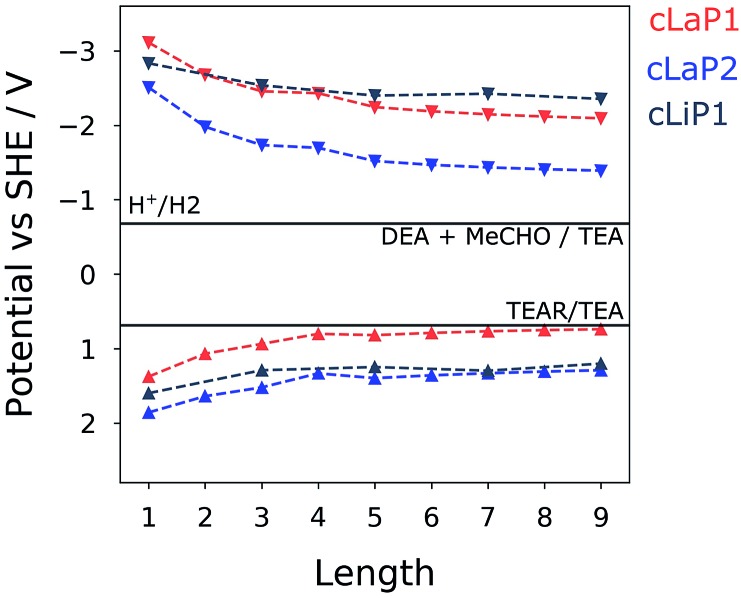
Predicted IP and EA values (*vs.* SHE) of various (ladder) polymers. IP and EA values have been computed for various oligomer lengths, where ‘length’ is equal to the number of aromatic rings along the polymer backbone. Hydrogen reduction and TEA oxidation potentials (pH = 11.5) are shown as horizontal lines.

To gain insights into changes in the thermodynamic driving force for proton reduction and TEA oxidation within the investigated series of polymers, the IP and EA levels as well as optical gaps for varying oligomer lengths (1–9; defined by the number of benzene bi-sulfoxide, thiophene or thiophene dioxide units, respectively) were estimated using a family of recently developed semi-empirical density functional tight-binding methods.^38^ Use of such methods, accompanied by a calibration procedure, has been shown to provide accurate optoelectronic properties with accuracy comparable to density functional theory.^39^ Different oligomer lengths were tested to ensure that converged values are obtained across both ladder and non-ladder polymer species. Fig. 7 shows the calculated IP and EA values compared to the hydrogen reduction and TEA oxidation potentials at pH = 11.5. We see that each of these polymers, both ladder and non-ladder, can create charge carriers with sufficient thermodynamic driving force to drive the necessary redox chemistry required for hydrogen evolution and TEA oxidation. Both **cLaP1**/**cLiP1** are predicted to have a larger driving force for proton reduction than **cLaP2**, while **cLaP2**/**cLiP1** have a larger driving force for overall TEA oxidation to diethylamine and acetaldehyde than **cLaP1**. Assuming that overall TEA oxidation can be effectively described as a combination of two subsequent one-hole transfer-steps to TEA species in solution (I: TEA + h^+^ → TEA^+^ → TEA radical (TEAR) + H^+^ and II: TEAR + h^+^ + H_2_O → diethylamine + acetaldehyde + H^+^), then the fact that **cLaP1** is predicted to have only negligible driving force for the first step, in contrast to **cLaP2**, might suggest that differences in catalytic activity between **cLaP1** and **cLaP2** stems from differences in the driving force for proton reduction. In line with experimental UV-vis absorption spectra, ladder polymers **cLaP1** and **cLaP2** are predicted to have optical gaps that are ∼1.0 and ∼0.8 eV lower for **cLaP1** and **cLaP2**, respectively, than for **cLiP1** (see Table S4†). Hence, **cLaP1** and **cLaP2** absorb more of the visible spectrum. The larger predicted optical gap of **cLaP1** relative to **cLaP2** (by approximately 0.1 eV) is also in line with experimental spectra.

## Discussion

The ladder polymer **cLaP1** outperforms its non-annulated parent polymer, **cLiP1**, significantly, and the optical properties of the material after annulation show a red-shift in the absorption onset. The ability of **cLaP1** to absorb more photons while maintaining a hydrogen reduction driving force at least partially explains its higher photocatalytic activity, especially under filtered visible light. No significant changes in the optical properties were observed upon oxidation of **cLaP1** to **cLaP2**, but the resulting dibenzo[*b*,*d*]thiophene sulfone material is almost inactive. This low activity of **cLaP2** is surprising since TCSPC shows that **cLaP2** has the longest weighted average lifetime of the exited state; significantly longer than those of **cLiP1** and **cLaP1**. Also, the introduction of dibenzo[*b*,*d*]thiophene sulfone motifs into other polymer photocatalysts has been reported to give materials with high photocatalytic activity.^8^,^24^,^25^,^31^ However, when taking the computationally predicted charge-carrier potentials into account, it becomes clear that **cLaP2** has a reduced overpotential for proton reduction relative to **cLaP1,** while **cLaP1** and **cLaP2** both have a reasonable driving force for TEA oxidation. From a thermodynamic perspective, **cLaP1** thus appears to be the best material in terms of combining thermodynamic driving force with light absorption. This is supported by TA measurements, which show the highest yield of long-lived charges in the case of **cLaP1**. From the TA data, it is clear that there is a direct correlation between the yield of long-lived charges present and the measured hydrogen evolution rate, suggesting that electron polaron states with lifetimes on the μs to ms timescale are required in order for hydrogen evolution to occur. This might rationalize the higher hydrogen evolution yields for **cLaP1**. The greater yield of long-lived photoelectrons may be related to more efficient hole scavenging at early timescales. The catalytic activity of the materials does not correlate with the residual palladium content, but it is unclear whether the threshold for an effect of the residual palladium on the photocatalytic performance has been reached.^9^,^34^ Since the TA spectrum of **cLaP2** shows a persistent long-lived feature, which could be attributed to a deep-trapped charge (Fig. 5, right), we loaded *in situ***cLaP1** and **cLaP2** with a platinum co-catalyst (1 wt%). In both cases, we observed an increase in photocatalytic performance, but there was no evidence for higher charge carrier yields in the TA spectrum of the platinized **cLaP2**.

In summary, the differences in photocatalytic activity in the series of **cLiP1**, **cLaP1** and **cLaP2** can be rationalized by comparison of charge carrier lifetimes, light absorption, and thermodynamic driving forces. Compared with related linear polymers, such as **P6**/**P60** and **P7**/**P10** (Fig. 2A), the idea of extending planarization across the full length of the polymer chain to enhance photocatalytic activity was not realized, at least not without the addition of a co-catalyst. For example, the photocatalytic hydrogen evolution rates for unmodified **cLaP1** (1307 μmol h^–1^ g^–1^) were found to be similar to those of its linear, non-ladderized analogues **P6** (1660 μmol h^–1^ g^–1^) and **P60** (1295 μmol h^–1^ g^–1^). However, when Pt was used as a co-catalyst, **cLaP1@Pt** outperformed **P60@Pt** under both broadband (2297 *vs.* 1703 μmol h^–1^ g^–1^) and visible light irradiation (1489 *vs.* 213 μmol h^–1^ g^–1^).

## Conclusions

Inspired by photocatalytically active dibenzo[*b*,*d*]thiophene and dibenzo[*b*,*d*]thiophene sulfone polymers, we set out to synthesize a new class of ladderized conjugated polymer photocatalysts for photocatalytic evolution of hydrogen from water. Through post-polymerization ladderization, a planarization of the polymer chain and expansion of the π-system could be achieved, as evidenced by the bathochromic shift of the absorption edge. A significant increase in photocatalytic activity was measured for one ladder polymer (**cLaP1**) while the other (**cLaP2**) remained almost inactive. The difference in photocatalytic activity could be rationalized by analysis of the charge carrier lifetimes *via* TA spectroscopy and comparison of the driving forces derived from calculations. These results suggest that post-polymerization ladderization could be a valuable technique in the preparation of efficient photocatalysts and that ladder polymers containing other photocatalytically active subunits might be considered for future studies.

## Conflicts of interest

There are no conflicts to declare.

## Supplementary Material

Supplementary information
