# Supplementary material for: Photocatalytically active ladder polymers
Source: Faraday Discuss. 2019 Jan 22;215:84–97. doi: 10.1039/c8fd00197a (PMC6677027; doi:10.1039/c8fd00197a)
Supplement: Supplementary file 1 [file FD-215-C8FD00197A-s001.pdf]

Electronic Supplementary Material (ESI) for Faraday Discussions.
This journal is © The Royal Society of Chemistry 2018

Supporting Information

for

Photocatalytically Active Ladder Polymers

Anastasia Vogel,[a] Mark Forster,[c] Liam Willbram,[b] Charlotte L. Smith,[a,c] Alexander Cowan,[c] Martijn A. Zwijnenburg,[b] Reiner Sebastian Sprick,[a] and Andrew I. Cooper[a,*]

Table of Contents

1 Experimental Section 3
2 UV-Vis and Photoluminescence Spectra 7
3 ATR-FT Infrared Spectroscopy 10
4 Powder X-Ray Diffraction 13
5 Thermogravimetric Analysis 15
6 Scanning Electron Microscope/Energy-dispersive X-ray spectroscopy 16
7 ICP-MS Measurements 17
8 Hydrogen Evolution Experiments for Polymers 18
9 External Quantum Efficiencies for **cLaP1** 21
10 Stability Test for **cLaP1** 22
11 Time-Correlated Single Photon Counting 24
12 Transient Absorption Spectroscopy 26
13 (GFN/IPEA/sTDA)-xTB Calculations 28
14 References 29

1 Experimental Section

Materials and methods:

All reagents and dry solvents were obtained from Sigma-Aldrich or from TCI and used as received. 1,4-Dibromo-2,5-bis(methylsulfinyl)benzene (**1**) was synthesized according to a previously published procedure.[1] Water for the hydrogen evolution experiments was purified using an ELGA LabWater system with a Purelab Option S filtration and ion exchange column without pH level adjustment. Reactions were carried out under nitrogen atmosphere using standard Schlenk techniques. CHN Analysis was performed on a Thermo EA1112 Flash CHNS-O Analyzer using standard microanalytical procedures. Palladium content was determined on a Perkin Elmer ICP-MS NexION 2000 using a Perkin Elmer Microwave Titan for digestion of powdered samples in conc. nitric acid. Transmission FT-IR spectra were recorded on a Bruker Alpha at room temperature using an ATR diamond sample tip. Thermogravimetric analysis was performed on a Q500 TGA by heating (20°C min^{-1}) samples under air (25 mL min^{-1}) in open platinum pans up to 1000 °C. The UV-visible absorption spectra were recorded on a Shimadzu UV-2550 UV-vis spectrometer as powders in the solid-state. The fluorescence spectra of the polymer powders were measured with a Shimadzu RF-5301PC fluorescence spectrometer at room temperature. Imaging of the polymer morphology was performed on a Hitachi S4800 Cold Field Emission SEM, with secondary electron, backscatter and transmission detectors. EDX Measurements were performed on an Oxford Instruments INCA ENERGY 250 M/X. PXRD Measurements were performed on a PANalytical X'Pert PRO MPD, with a Cu X-ray source, used in high throughput transmission mode with Kα focusing mirror and PIXCEL 1D detector. Time-correlated single photon counting experiments (TCSPC) were performed on an Edinburgh Instruments LS980-D2S2-STM spectrometer equipped with picosecond pulsed LED excitation sources and a R928 detector, with a stop count rate below 3%. A 371.5 nm laser diode (instrument response 100 ps fwhm) was used. Suspensions were prepared by ultrasonicating the polymers in water (concentration is the same as used in the corresponding TA experiments). The instrument response was measured with colloidal silica (LUDOX® HS-40, Sigma-Aldrich) at the excitation wavelength and decay times were fitted in Fluoracle software, based on suggested lifetime estimates and pre-exponential factors.

Hydrogen evolution experiments:

A screw-top vial was charged with the polymer powder (*ca.* 1 mg mL^{-1}) and a mixture of water/triethylamine/MeOH (1:1:1; 10 mL) and ultrasonicated until the photocatalyst was dispersed (30 min). The suspension was transferred into a quartz cuvette (2 × 4 × 1 cm, w × h × d), sealed with a septum and degassed by N_2 bubbling for 30 min. The reaction mixture was illuminated with a 300 W Newport Xe light-source (Model: 6258, Ozone free) for the time specified using appropriate filters. Gas samples were taken with a gas-tight syringe, and run on a Bruker 450-GC gas chromatograph equipped with a Molecular Sieve 13X 60-80 mesh 1.5 m × ⅛" × 2 mm ss column at 50°C with an argon flow of 40.0 mL min^{-1}. Hydrogen was detected with a thermal conductivity detector referencing against standard gas with a known concentration of hydrogen. Hydrogen dissolved in the reaction mixture was not measured and the pressure increase generated by the evolved hydrogen was neglected in the calculations. The rates were determined from a linear regression fit (see Section 8 for fits) and the error is given as the standard deviation of the amount of hydrogen evolved. No hydrogen evolution was observed for a mixture of water/methanol/trimethylamine under λ >295 nm illumination in absence of a photocatalyst.

External quantum efficiency measurements:

The external quantum efficiencies of the polymer photocatalysts for hydrogen evolution were estimated using monochromatic light from LEDs controlled by an IsoTech IPS303DD power supply. The output of the LEDs was measured with a ThorLabs S120VC photodiode power sensor controlled by a ThorLabs PM100D Power and Energy Meter Console to be 1.80 mW for the λ = 375 nm LED, 3.18 mW for the λ = 420 nm LED, 1.95 mW for the λ = 515 nm LED, and 5.05 mW for the λ = 595 nm LED. For the experiments polymer (8 mg) was suspended in water, triethylamine, methanol (1:1:1 volume mixture) and an area of 8 cm^2 was illuminated (path length = 1 cm) and the amount of hydrogen was measured as described above. For **cLaP1@Pt** the sample was prepared, H_2PtCl_6 solution was added and illuminated for 1 hour under λ > 295 nm illumination (300 W Xe light source). The sample was then degassed again and illuminated using a λ = 420 nm LED. The external quantum efficiencies were estimated using the equation below:

$$EQE_{\lambda} = 2 \times \frac{moles\ of\ hydrogen\ evolved}{moles\ of\ incident\ photons} \times 100\%$$

Synthesis of **cLiP1**:[2]

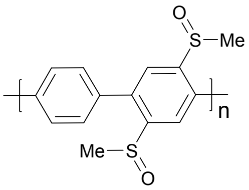

1,4-Dibromo-2,5-bis-(methylsulfinyl)benzene (1.00 g, 2.78 mmol), 1,4-phenylene bis(pinacol boronate) (918 mg, 2.78 mmol), $NaHCO_3$ (1.70 g, 42 mmol), $NaHCO_3$ aqueous solution (saturated, 4.5 mL), 1,2-dimethoxyethane (10 mL), and $[Pd(PPh_3)_4]$ (324 mg, 0.28 mmol) were combined in a three-necked round-bottom flask (50 mL). The solution was degassed by bubbling nitrogen through (5 min), and then the reaction mixture was heated to 90 °C and stirred for 24 h. After cooling to room temperature, H_2O (250 mL) was added and the solution was extracted with chloroform (3 × 250 mL). The organic layer was dried over anhydrous Na_2SO_4, filtered and evaporated to dryness. The crude oligomer was purified by precipitation into methanol (600 mL) from hot $CHCl_3$ solution (200 mL). After drying in a vacuum overnight at room temperature, the oligomer **cLiP1** was obtained as an off-white powder (412 mg, 54 %).

Anal. Calcd for $(C_{14}H_{12}O_2S_2)_n$: C, 60.84; H, 4.38%; Found C, 53.15; H, 4.15%.

Synthesis of **cLaP1**:[2]

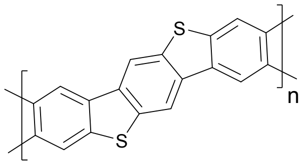

Oligomer **cLiP1** (400 mg) was added to cold trifluoromethanesulfonic acid (40 mL) in a round-bottom flask (100 mL) and stirred at room temperature for 22 h. The mixture was added to cold water (500 mL) with vigorous stirring to precipitate the product. The precipitate was filtered and washed with water several times. The methylated oligomer **cLaP1$^+$-Me** (336 mg) was obtained as a light brown powder, after drying in a vacuum overnight at room temperature.

The methylated oligomer **cLaP1⁺-Me** (336 mg) was dissolved in a mixture of acetone/acetonitrile (80 mL, 1:1) at room temperature. Tetraethylammonium bromide (1.30 g) dissolved in an acetonitrile (10 mL) and water (2 mL) mixture was added to the oligomer **cLaP1⁺-Me** solution and stirred vigorously at room temperature for 18 h. The yellow solid oligomer **cLaP1⁺-Me** (223 mg), which precipitated as the result of demethylation, was collected by filtration, washed with water, and dried overnight at room temperature.

Oligomer **cLaP1⁺-Me** (223 mg) was added to cold trifluoromethanesulfonic acid (15 mL) in a round-bottom flask and the reaction mixture was stirred at room temperature for 21 h. The reaction solution was poured into cold water (300 mL) with vigorous stirring to precipitate the product. The precipitate was filtered and washed with water several times. Oligomer **cLaP1** was obtained as a brown powder (222 mg, 65%), after drying in a vacuum overnight at room temperature.

Anal. Calcd for $(C_{12}H_4S_2)_n$: C, 67.90; H, 1.90%; Found C, 48.35; H, 2.76%.

Synthesis of **cLaP2**:[3]

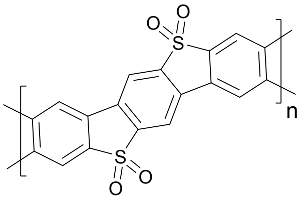

Oligomer **cLaP1**(100 mg) was dissolved in acetic acid (glacial, 5 mL) and heated to reflux (approx. 130 °C). Hydrogen peroxide solution (aqueous, 30 wt. %, 0.5 mL) was added dropwise and the reaction mixture was heated under reflux for 4 h. The reaction mixture was cooled down to room temperature and poured into cold DI water (50 mL). The precipitate was collected and washed with DI water. The product **cLaP2** (93 mg, 79%) was dried at room temperature *in vacuo* overnight.

Anal. Calcd for $(C_{12}H_4O_4S_2)_n$: C, 52.17; H, 1.46%; Found C, 45.46; H, 2.61%.

Synthesis of 3,7-bis(4,4,5,5-tetramethyl-1,3,2-dioxaborolan-2-yl)dibenzo[*b*,*d*]thiophene:[4]

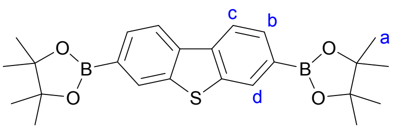

Under inert conditions, a flask was charged with 3,7-dibromodibenzo[*b,d*]thiophene (1 g, 2.9 mmol, 1 eq), B_2Pin_2 (1.6 g, 6.4 mmol, 2.2 eq.) and $[Pd(Cl)_2(dppf)_2]$ (64 mg, 3 mol%). Anhydrous DMSO (40 ml) and KOAc (1.7 g, 6 eq.) were added. The reaction mixture was heated to 80 °C for 28 hours. After cooling to room temperature, the mixture was poured onto ice and the precipitate was collected via filtration. The crude product was re-dissolved in ethyl acetate (120 ml) and filtered through a celite pad. The solvent was removed under reduced pressure. The beige product (965.2 mg, 75%.) was dried *in vacuo* at 40 °C for 48 hours.

IR (ATR, cm^{-1}): $\tilde{\nu}$ = 3053 (w),2977 (m),2925 (w),1736 (w), 1591 (m), 1545 (w), 1472 (m), 1388 (s),1369 (s), 1344 (vs), 1269 (s),1255 (s),1209 (m), 1165 (m), 1135 (vs), 1094 (vs), 1067 (m), 966 (s), 859 (s), 840 (s), 817 (s), 721 (s), 664 (s), 643 (m), 577 (m), 522 (m), 414 (m).

^{1}H NMR (300 MHz, $CDCl_3$, 298 K): δ (ppm) = 8.34 (dd, J = 0.8, 0.3 Hz, 2H, H^d), 8.20 (dd, J = 7.9, 0.3 Hz, 2H, H^c), 7.88 (dd, J = 7.9, 0.8 Hz, 2H, H^b), 1.39 (s, 24H, $CH_3{}^a$).

Synthesis of **P60**:[5]

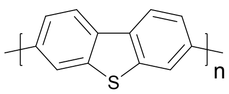

A flask was charged with 3,7-dibromodibenzo[*b*,*d*]thiophene (257 mg, 0.75 mmol), 3,7-bis-(4,4,5,5-tetramethyl-1,3,2-dioxaborolan-2-yl)dibenzo[*b*,*d*]thiophene (327 mg, 0.75 mmol), *N*,*N*-dimethylformamide (20 mL), an degassed, aqueous solution of K_2CO_3 (4 mL, 2.0 M), and $[Pd(PPh_3)_4]$ (15 mg, 2 mol%). After cooling to room temperature, the mixture was poured into water (150 mL). The precipitate was collected by filtration and washed with H_2O (50 mL) and methanol (100 mL). The crude product was purified by Soxhlet extraction with chloroform for 22 h. The beige product (236 mg, 86%) was collected and dried *in vacuo* at 80°C overnight.

Anal. Calcd for $(C_{24}H_{12}S_2)_n$: C, 79.09; H, 3.32%; Found C, 71.42; H, 3.45%.

2 UV-Vis and Photoluminescence Spectra

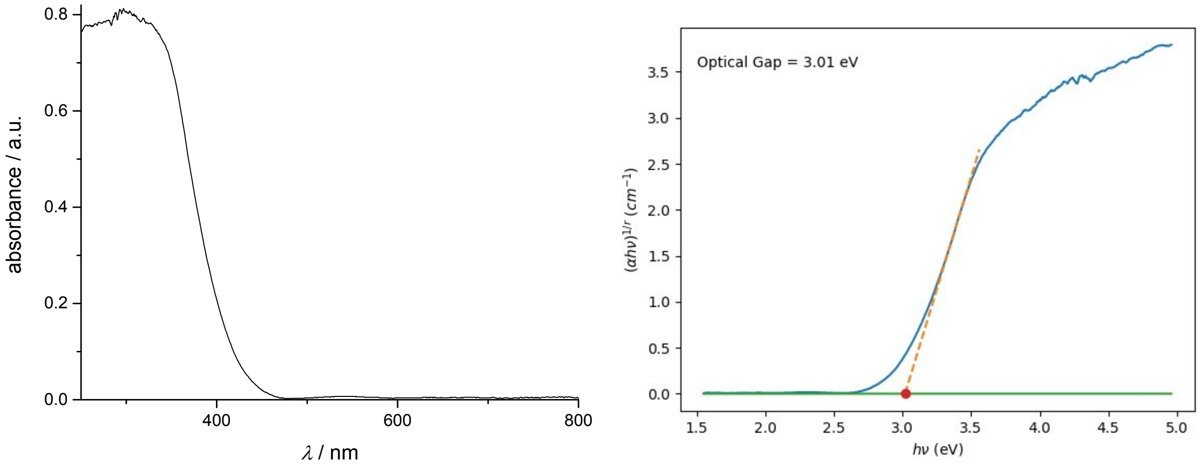

*Figure S1. Solid-state UV-vis spectrum (left) and Tauc plot[6,7] with estimated optical gap (right) for **cLiP1**.*

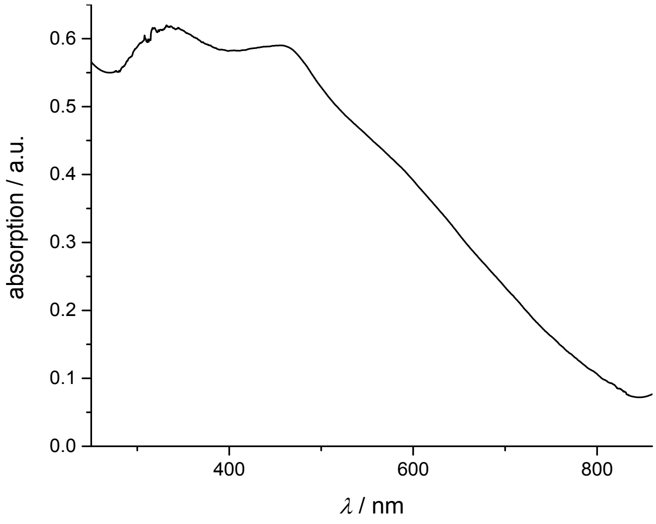

*Figure S2. Solid-state UV-vis spectrum for **cLaP1⁺-Me**.*

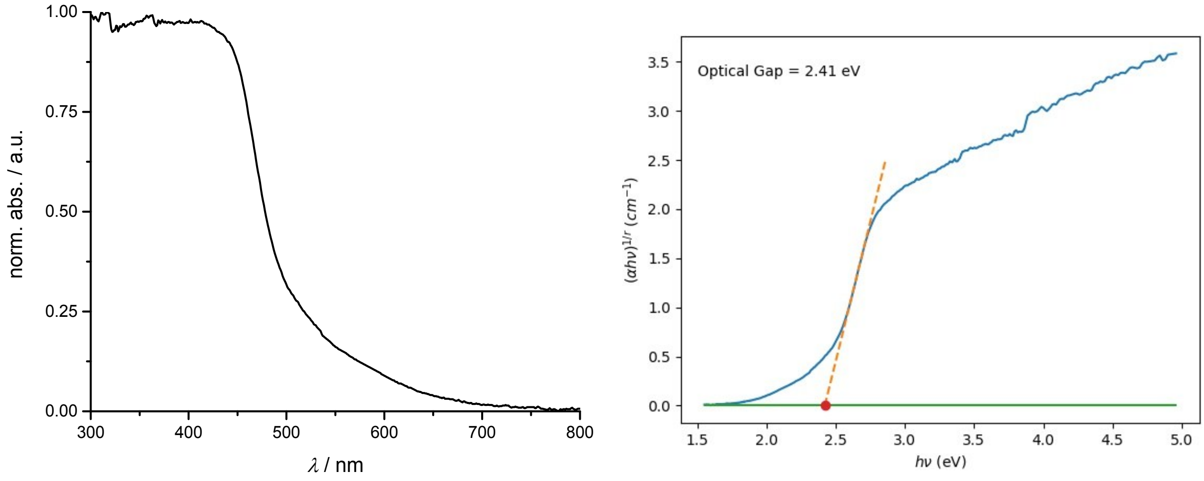

*Figure S3. Solid-state UV-vis spectrum (left) and Tauc plot[6,7] with estimated optical gap (right) for **cLaP1**.*

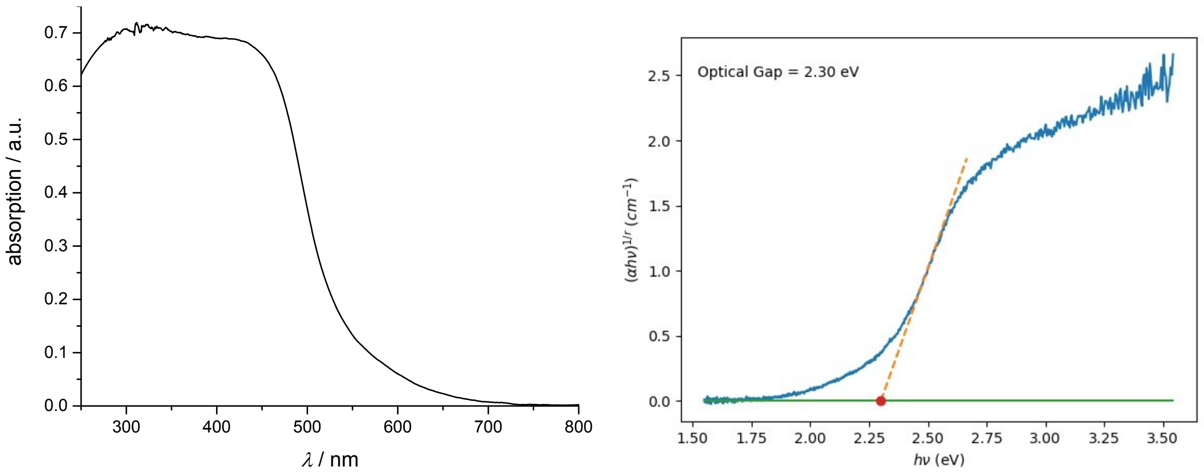

*Figure S4. Solid-state UV-vis spectrum (left) and Tauc plot with estimated optical gap (right) for **cLaP2**.*

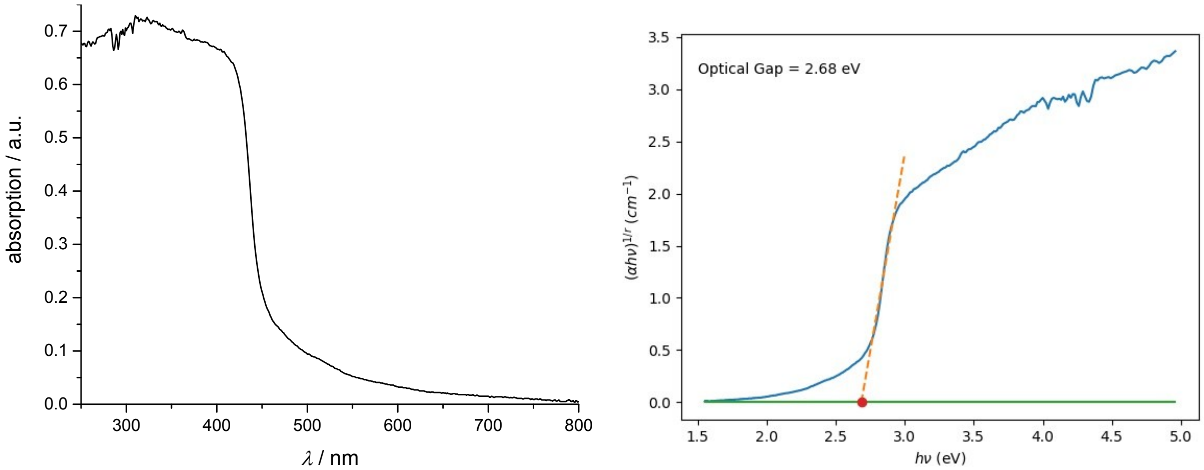

*Figure S5. Solid-state UV-vis spectrum (left) and Tauc plot with estimated optical gap (right) for **P60**.*

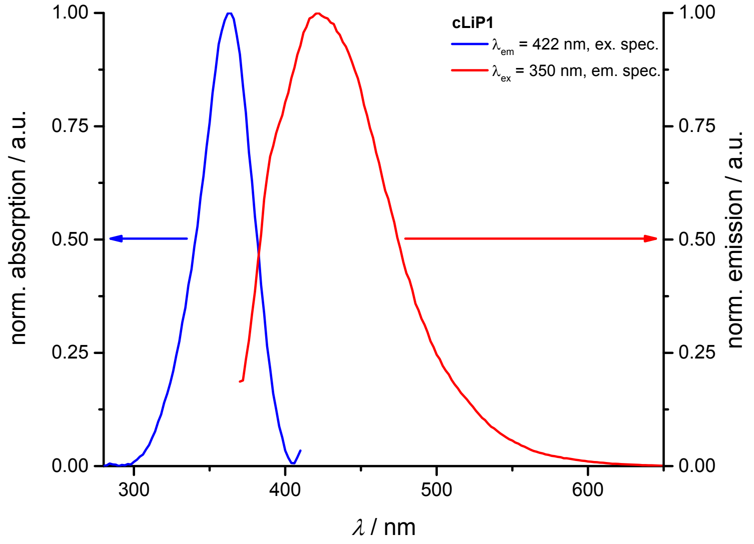

*Figure S6. Excitation and emission spectra for **cLiP1**.*

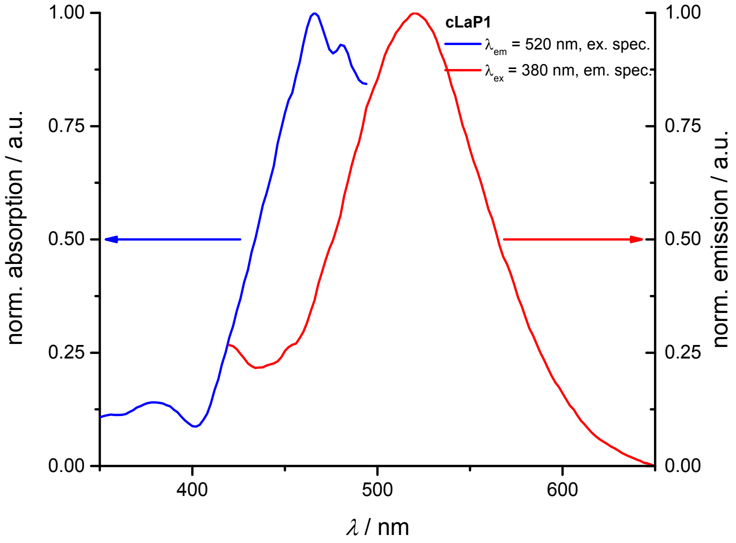

Figure S7.Excitation and emission spectra for ***cLaP1****.*

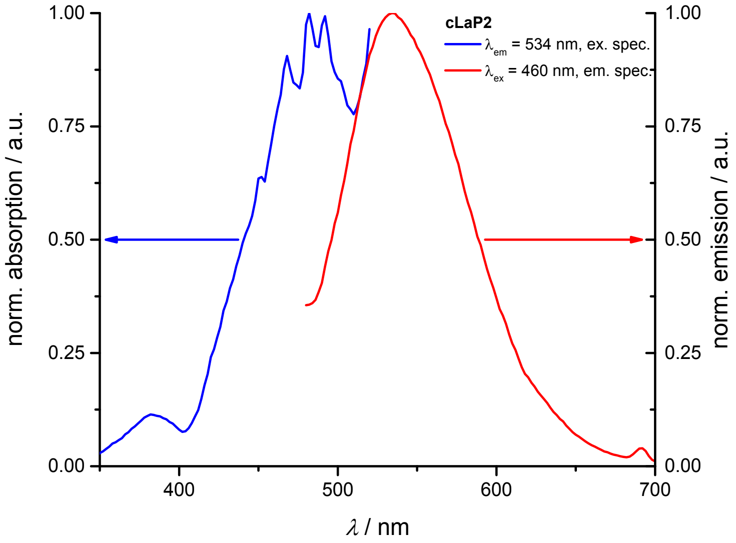

Figure S8. Excitation and emission spectra for ***cLaP2****.*

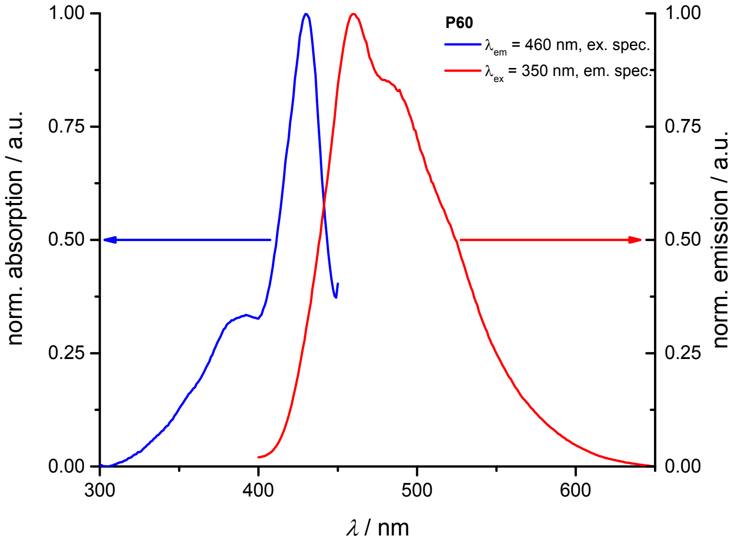

Figure S 9. Excitation and emission spectra for ***P60****.*

3 ATR-FT Infrared Spectroscopy

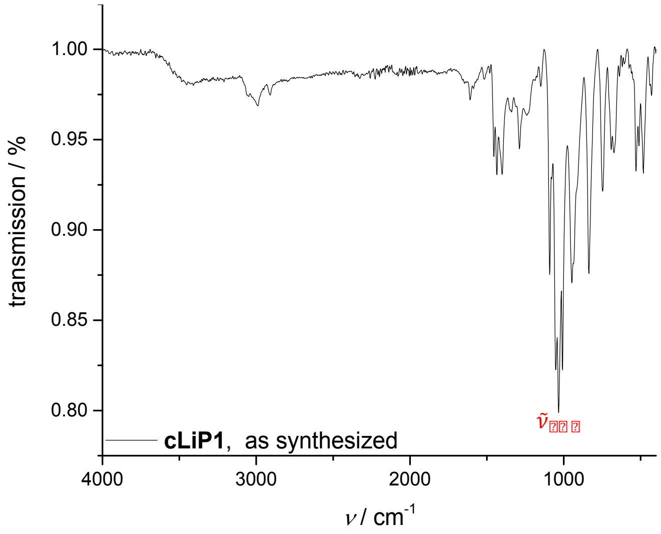

*Figure S10. ATR FTIR spectrum for **cLiP1** as synthesized with assignment of most prominent bands.*

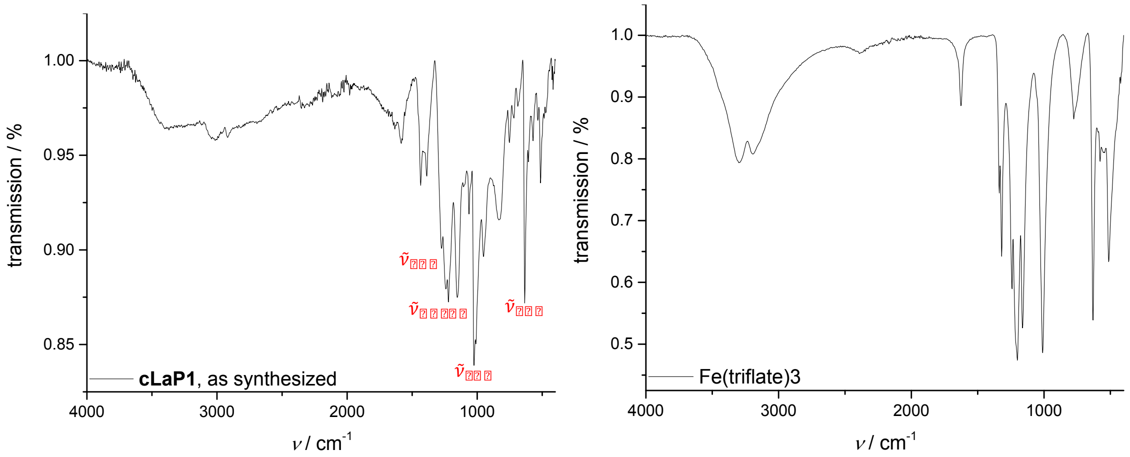

*Figure S11. ATR FTIR spectrum for **cLaP1** (left) as synthesized with assignment of most prominent bands and ATR FTIR spectrum for Fe(triflate)$_3$ for comparison (right).*

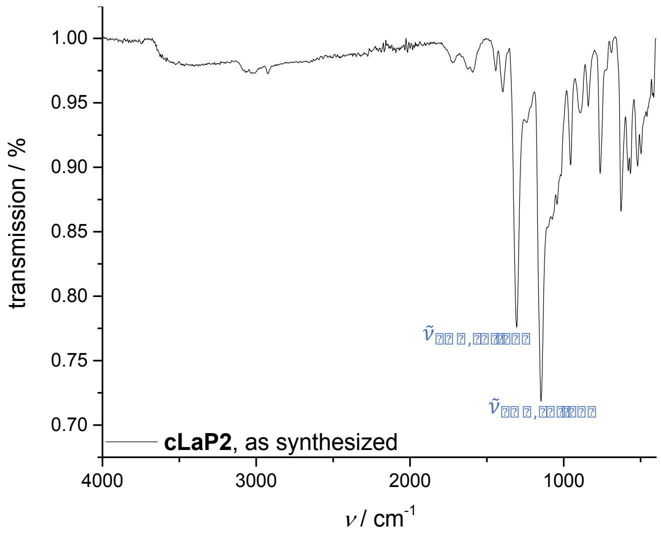

*Figure S12. ATR FTIR spectrum for **cLiP2** as synthesized with assignment of most prominent bands.*

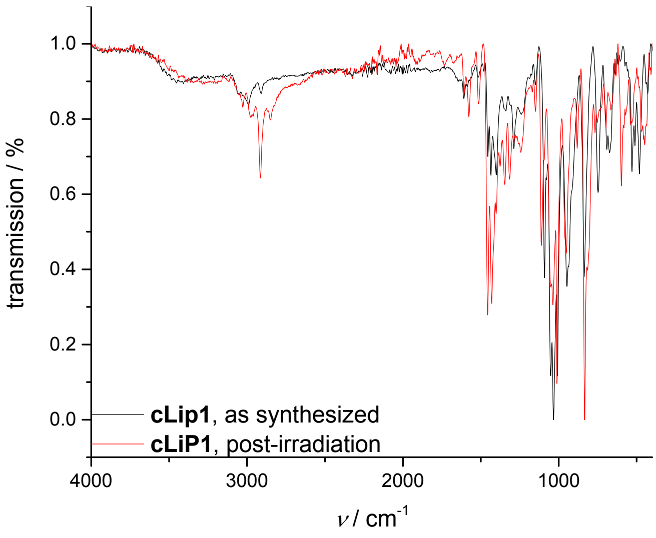

*Figure S13. ATR FTIR spectrum comparison for **cLiP1** as synthesized (black) and recovered after catalysis (red).*

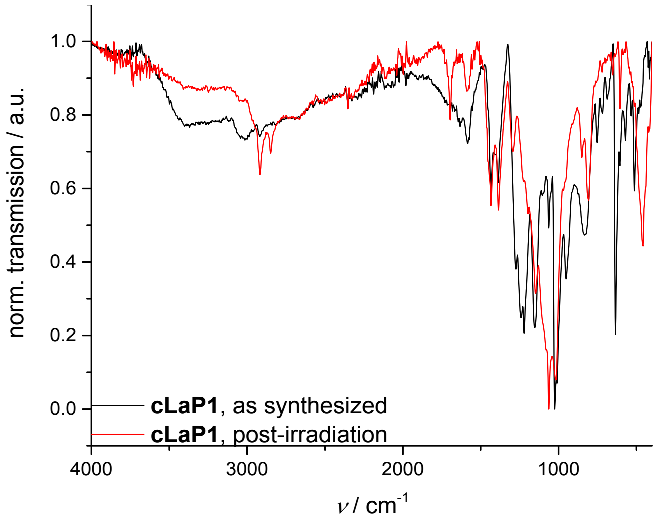

*Figure S14. ATR FTIR spectrum comparison for **cLaP1** as synthesized (black) and recovered after catalysis (red).*

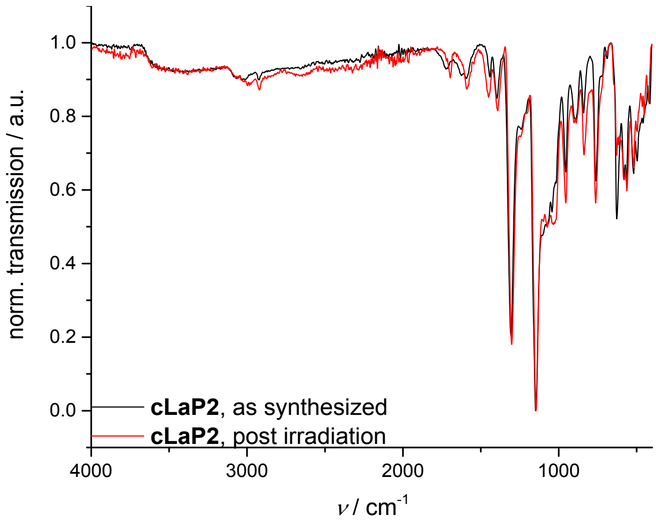

*Figure S15. ATR FTIR spectrum comparison for **cLaP2** as synthesized (black) and recovered after catalysis (red).*

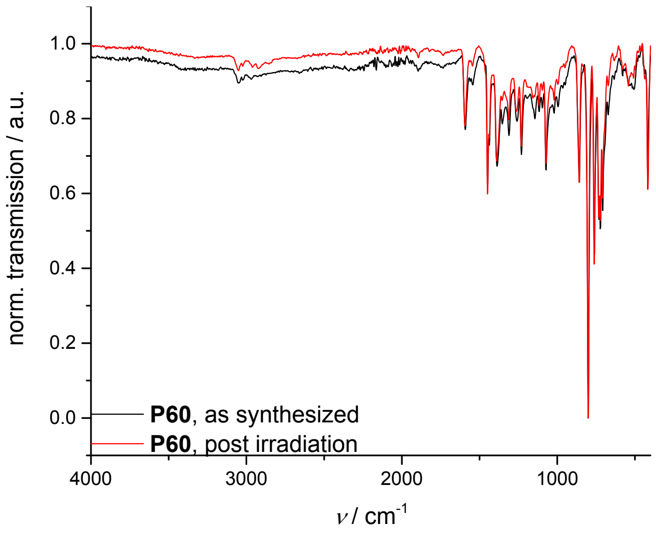

*Figure S16. ATR FTIR spectrum comparison for **P60** as synthesized (black) and recovered after catalysis (red).*

4 Powder X-Ray Diffraction

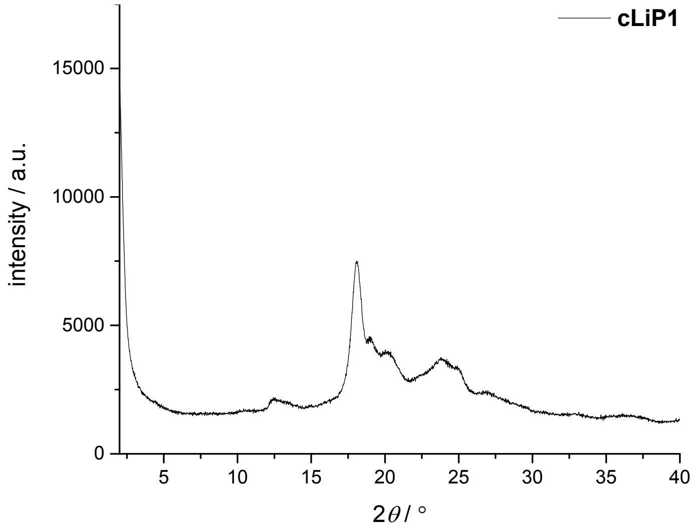

*Figure S17. PXRD pattern of **cLiP1**.*

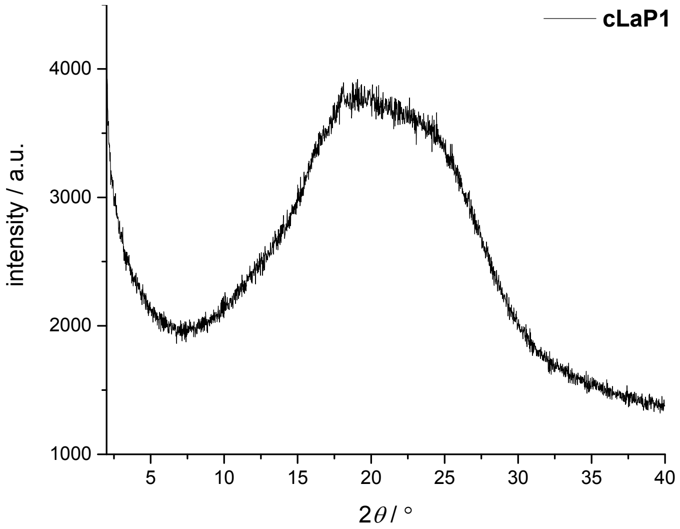

*Figure S18. PXRD pattern of **cLaP1**.*

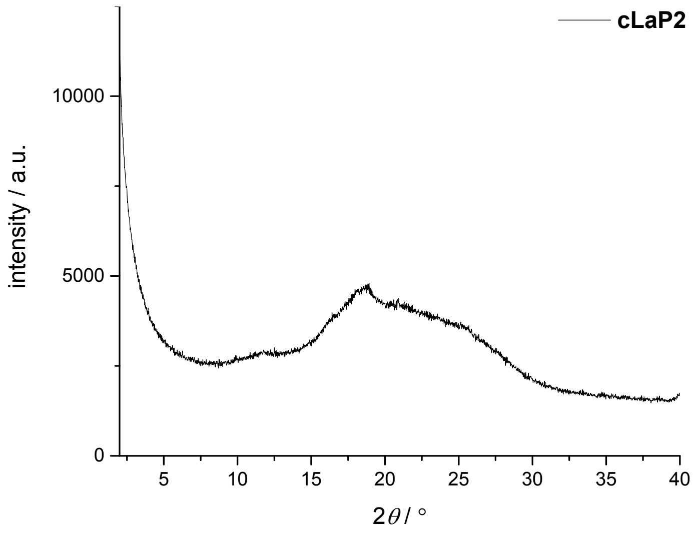

*Figure S19. PXRD pattern of **cLaP2**.*

5 Thermogravimetric Analysis

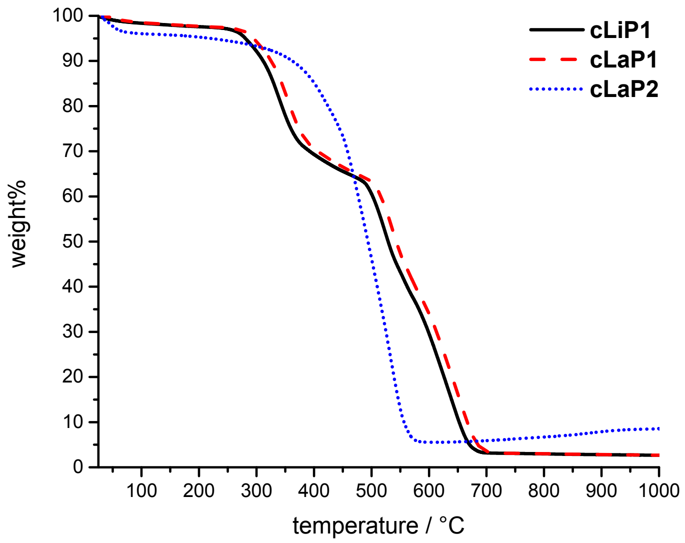

*Figure S20.TGA traces for **cLiP1**, **cLaP1** and **cLaP2**.*

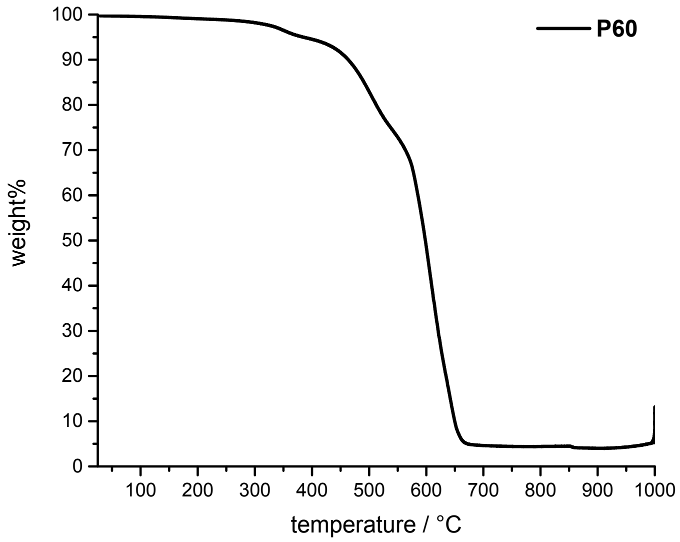

*Figure S21. TGA trace for **P60**.*

6 Scanning Electron Microscope/Energy-dispersive X-ray spectroscopy

cLiP1

cLaP1

cLaP2

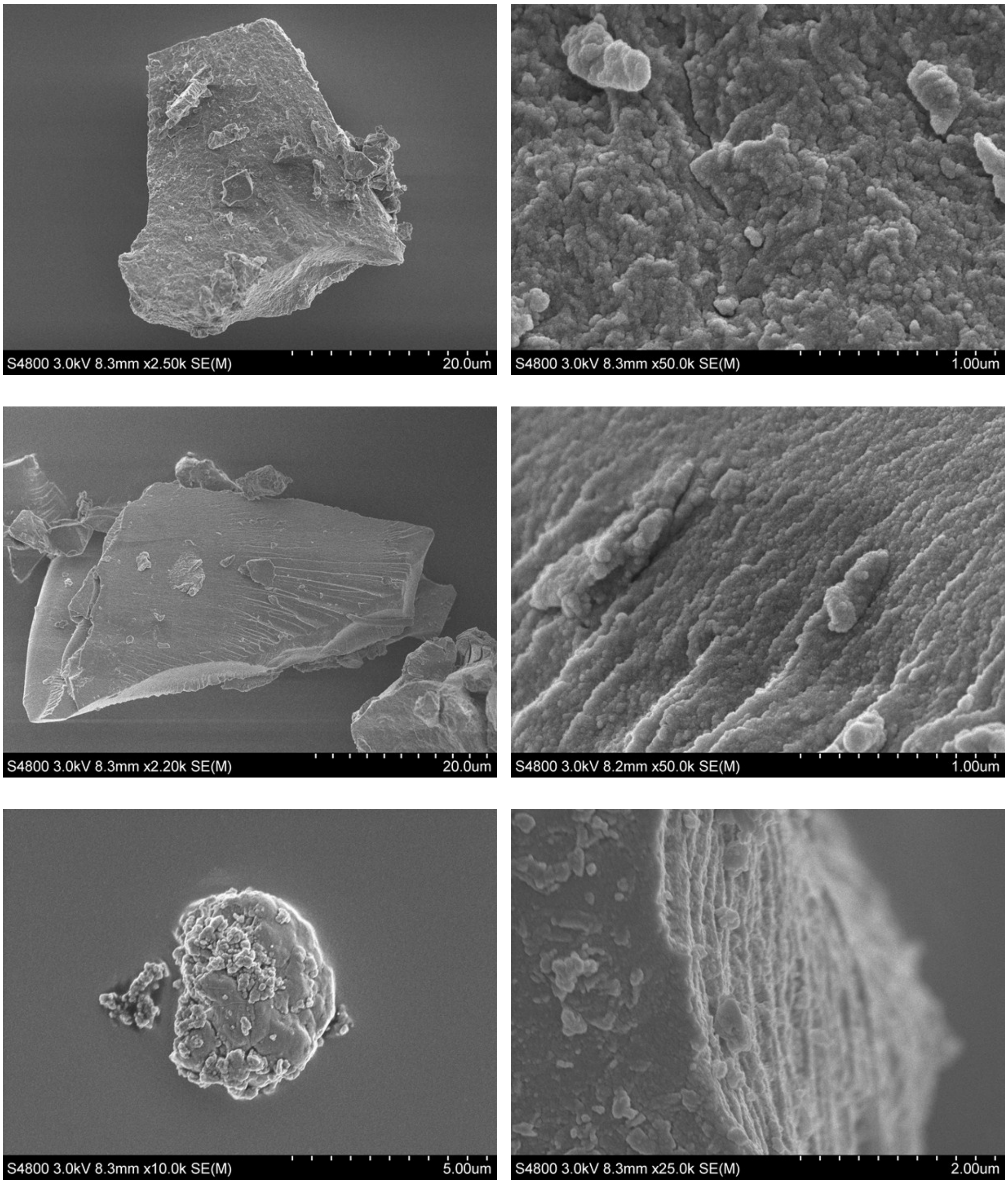

*Figure S22. SEM images for **cLiP1**, **cLaP1** and **cLaP2**.*

Table S1. Energy-dispersive X-ray spectroscopy. Average apparent composition of the sample determined via energy-dispersive X-ray spectroscopy in at least two points of the sample.

Element	cLiP1 / avg. wt. %	cLaP1 / avg. wt. %	cLaP2 / avg. wt. %
C	50.39	34.25	35.52
S	19.13	35.21	25.57
O	23.14	17.72	31.97
Br	5.33	7.85	4.87
Pd	0.88	0.79	0.42
P	0.62	0.78	0.63

7 ICP-MS Measurements

Table S2. Average palladium content in samples.

Sample	wt. %
**P60**	0.49
**cLiP1**	0.83
**cLaP1**	0.38
**cLaP2**	0.36

8 Hydrogen Evolution Experiments for Polymers

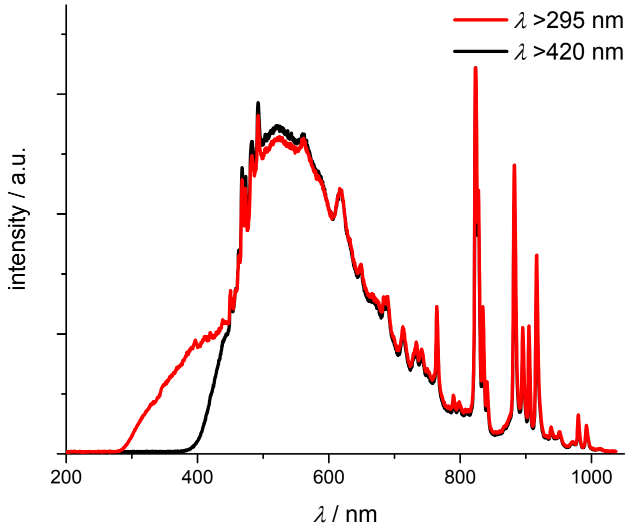

Figure S23. Output profile of the 300 W Xe-lamp equipped with a >295 nm (red) or a >420 nm (black) filter.

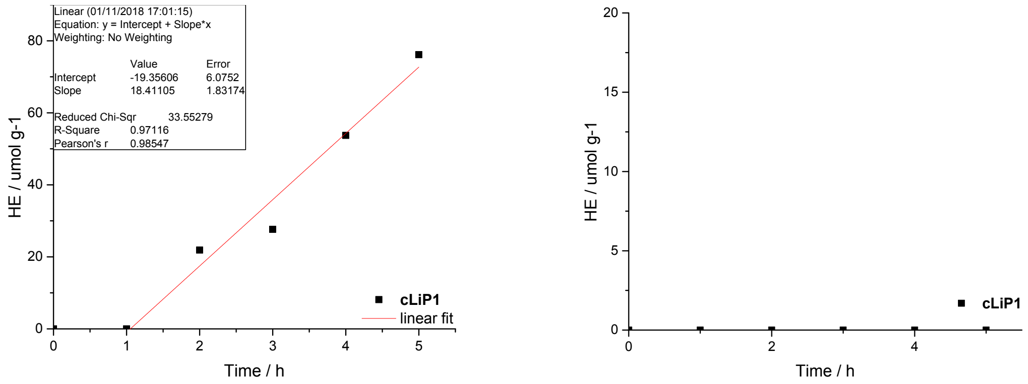

*Figure S24. Hydrogen evolution of **cLiP1** from a triethylamine/water/methanol mixture under λ >295 nm (left) and λ >420 nm (right) irradiation.*

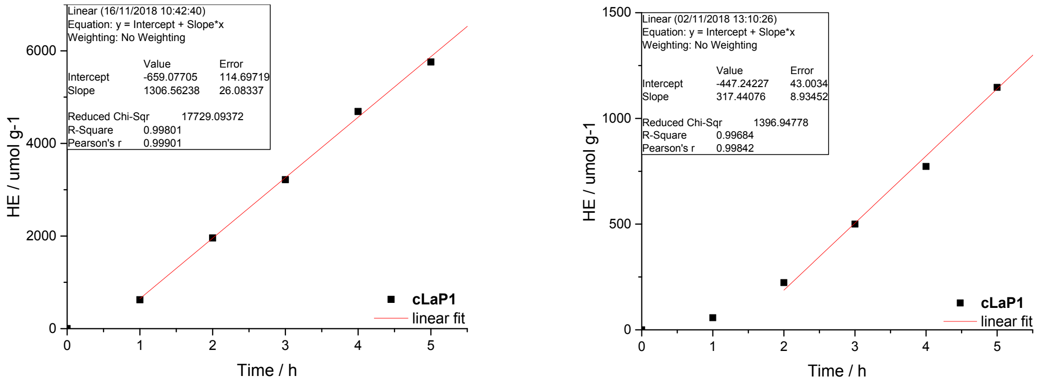

*Figure S25. Hydrogen evolution of **cLaP1** from a triethylamine/water/methanol mixture under λ >295 nm (left) and λ >420 nm (right) irradiation.*

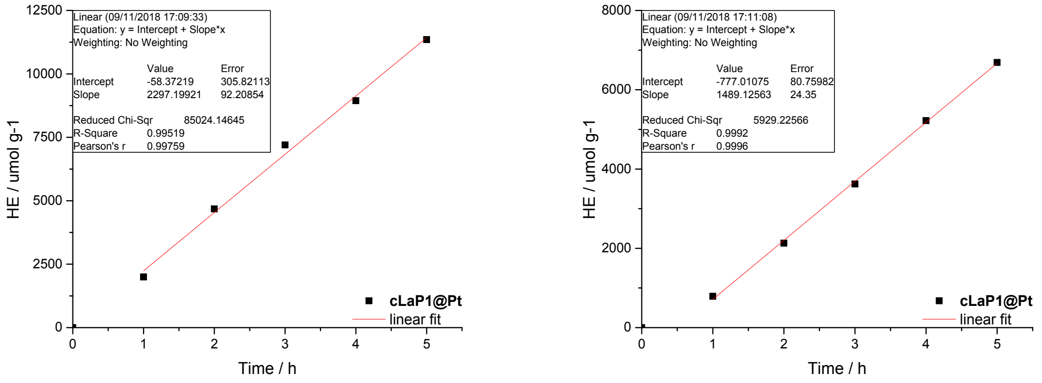

*Figure S26. Hydrogen evolution of **cLiP1@Pt** from a triethylamine/water/methanol mixture under λ >295 nm (left) and λ >420 nm (right) irradiation.*

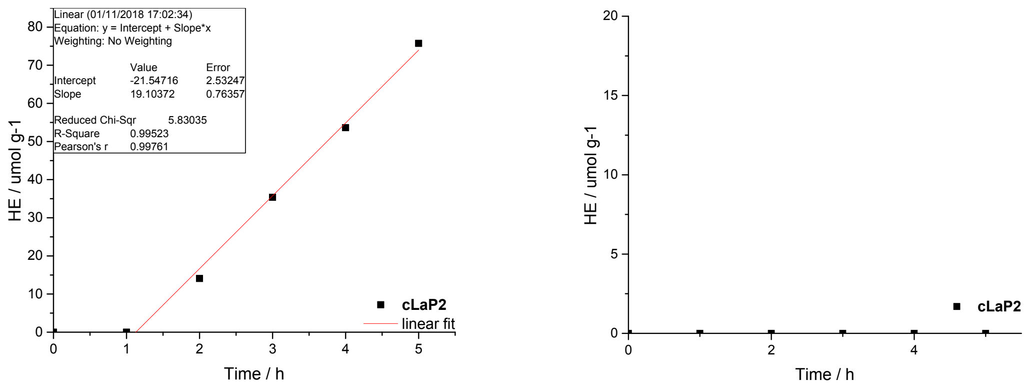

*Figure S27. Hydrogen evolution of **cLaP2** from a triethylamine/water/methanol mixture under λ >295 nm (left) and λ >420 nm (right) irradiation.*

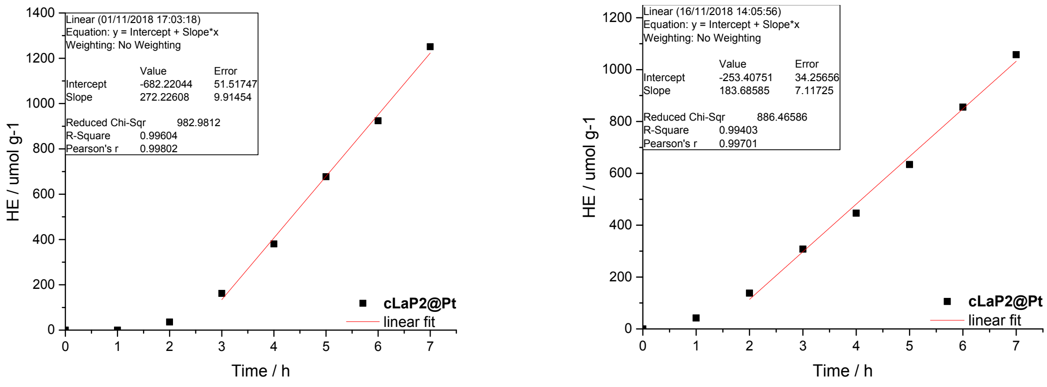

*Figure S28. Hydrogen evolution of **cLaP2@Pt** from a triethylamine/water/methanol mixture under λ >295 nm (left) and λ >420 nm (right) irradiation.*

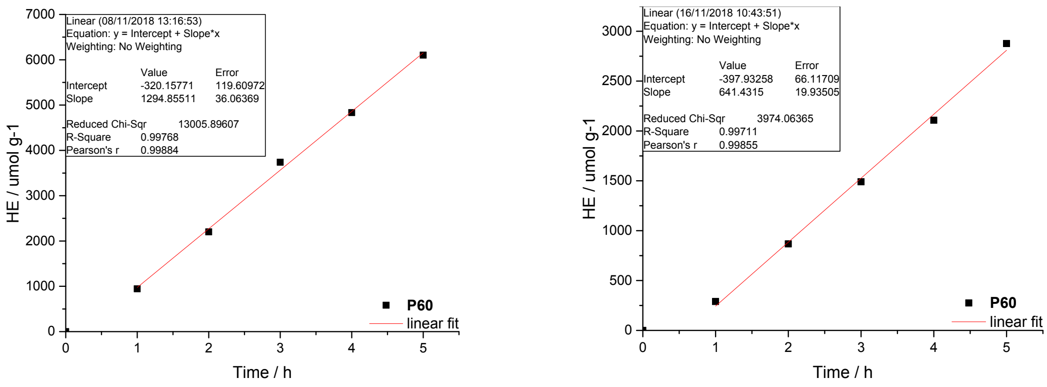

*Figure S29. Hydrogen evolution of **P60** from a triethylamine/water/methanol mixture under λ >295 nm (left) and λ >420 nm (right) irradiation.*

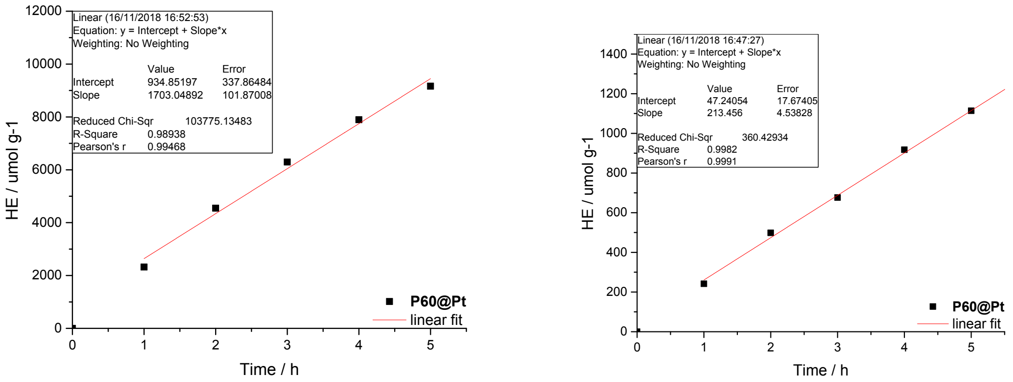

*Figure S30. Hydrogen evolution of **P60@Pt** from a triethylamine/water/methanol mixture under λ >295 nm (left) and λ >420 nm (right) irradiation.*

9 External Quantum Efficiencies for **cLaP1**

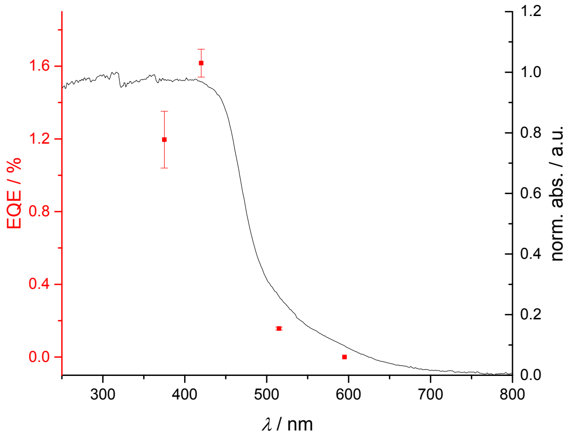

*Figure S31. External quantum efficiencies of **cLaP1** (8 mg) from a triethylamine/water/methanol mixture (8 mL) at 375, 420, 515 and 595 nm (±10 nm, fwhm LEDs).*

10 Stability Test for **cLaP1**

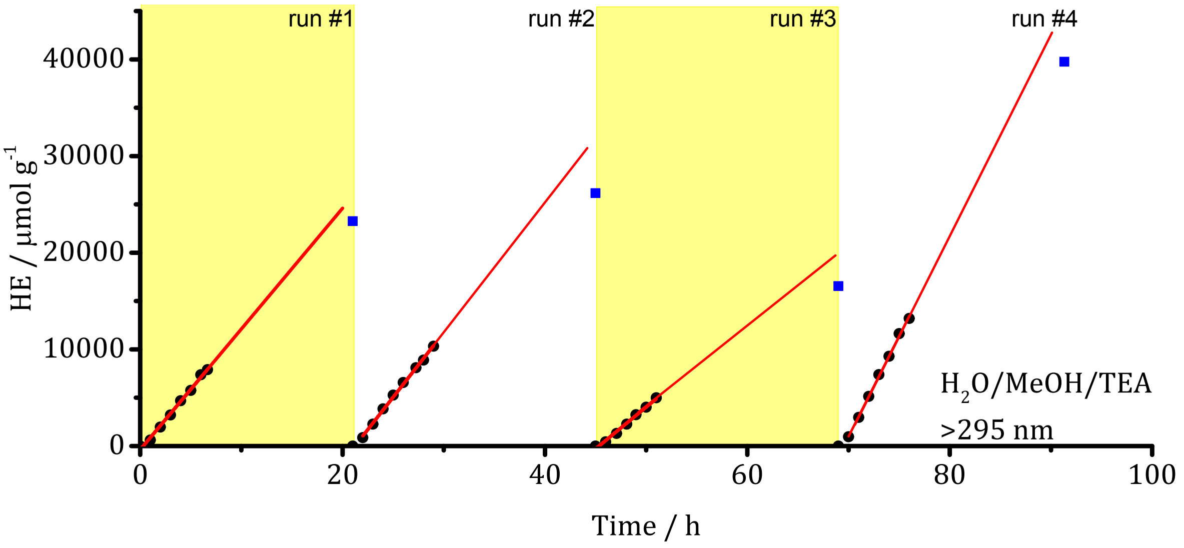

*Figure S32. Long-term catalytical run (90 hours) for **cLaP1**.*

	Irradiation conditions and sample treatment
**run #1**	8.3 mg, H2O/TEA/MeOH (1:1:1; 10 mL), degassed by bubbling N_2, >295 nm cut-off filter, 300 W Xe lamp;<br><br>Sample was irradiated for 7 hours, samples of the gas phase were taken hourly (black circles) and the hydrogen evolution rate was estimated via a linear fit function (red line). Sample was left irradiated overnight. A final sample of the gas phase was taken the next morning (blue square) to ensure that hydrogen evolution had continued overnight. Slight deviation from the linear fit function is expected due to increase in pressure and possible leakage through the punctured septum.<br>Estimated HER: 1307 ± 26 µmol h^{-1} g^{-1}
	Between run #1 and run #2, septum was exchanged and the sample was degassed.
**run #2**	sample from run #1, >295 nm cut-off filter, 300 W Xe lamp;<br><br>Sample was irradiated for 8 hours, samples of the gas phase were taken hourly (black circles) and the hydrogen evolution rate was estimated via a linear fit function (red line). Sample was left irradiated overnight. A final sample of the gas phase was taken the next morning (blue square) to ensure that hydrogen evolution had continued overnight. Slight deviation from the linear fit function is expected due to increase in pressure and possible leakage through the punctured septum.<br>Estimated HER: 1348 ± 34 µmol h^{-1} g^{-1}
	Between run #2 and #3, septum was exchanged and the sample was degassed.
**run #3**	sample from run #2, >295 nm cut-off filter, 300 W Xe lamp;<br><br>Sample was irradiated for 6 hours, samples of the gas phase were taken hourly (black circles) and the hydrogen evolution rate was estimated via a linear fit function (red line). Sample was left irradiated overnight. A final sample of the gas phase was taken the next morning (blue square) to ensure that hydrogen evolution had continued overnight. Slight deviation from the linear fit function is expected due to increase in

pressure and possible leakage through the punctured septum. A lower hydrogen evolution rate was estimated. We attribute this to an exhaustion of the sacrificial donor mixture and possible “poisoning” due to accumulation of byproducts.
Estimated HER: 921 ± 17 μmol h^{-1} g^{-1}

Between run #3 and #4, sample was recovered from the reaction mixture by centrifugation, removal of the supernatant, re-dispersion in H_2O (10 mL), repeated centrifugation and final filtration. Recovered **cLaP1** (5.0 mg) was dried *in vacuo*. A sample was taken for IR and UV-vis measurements at this point.

---

run #4 5.0 mg, H2O/TEA/MeOH (1:1:1; 10 mL), >295 nm cut-off filter, 300 W Xe lamp;

Sample was irradiated for 7 hours, samples of the gas phase were taken hourly (black circles) and the hydrogen evolution rate was estimated via a linear fit function (red line). Sample was left irradiated overnight. A final sample of the gas phase was taken the next morning (blue square) to ensure that hydrogen evolution had continued overnight. Slight deviation from the linear fit function is expected due to increase in pressure and possible leakage through the punctured septum. A higher hydrogen evolution rate was estimated. We attribute this to a lower effective mass of the recovered **cLaP1** in comparison to still partially methylated initial catalyst as yielded from synthesis.
Estimated HER: 2078 ± 34 μmol h^{-1} g^{-1}

---

11 Time-Correlated Single Photon Counting

Fluorescence life-times are obtained from fitting time-correlated single photon counting decays to a sum of three exponentials, which yield τ_1, τ_2 and τ_3, according to Equation 1.

Equation 1.Fitting function for time-correlated single photon counting decays.

$$\sum_{i=1}^{n}\left(A + B_i\,exp(-t/\tau_i)\right)$$

Weighted average lifetimes τ_{av} are calculated according to Equation 2.

Equation 2. Calculation of weighted average lifetime.

$$\sum_{i=1}^{n} B_i \tau_i$$

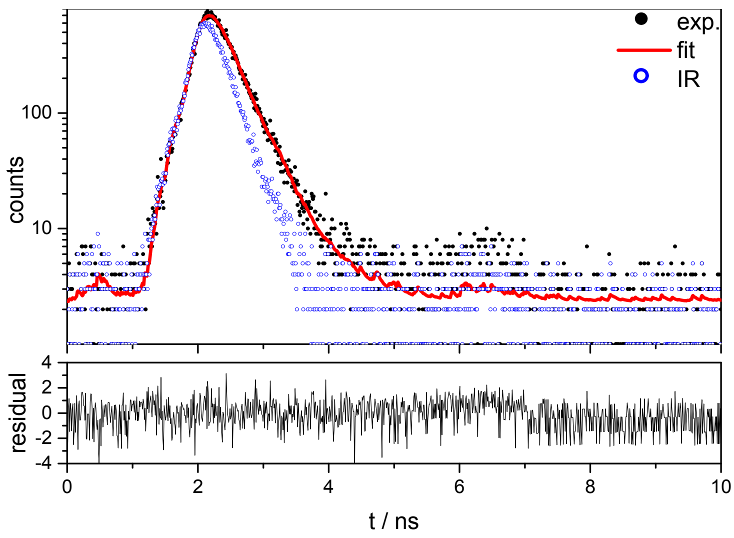

*Figure S33. Fluorescence life-time decay of **cLiP1** in aqueous suspension; instrument response (IR) shown in blue circles, experimental data in black dots and fitting function as a red line.*

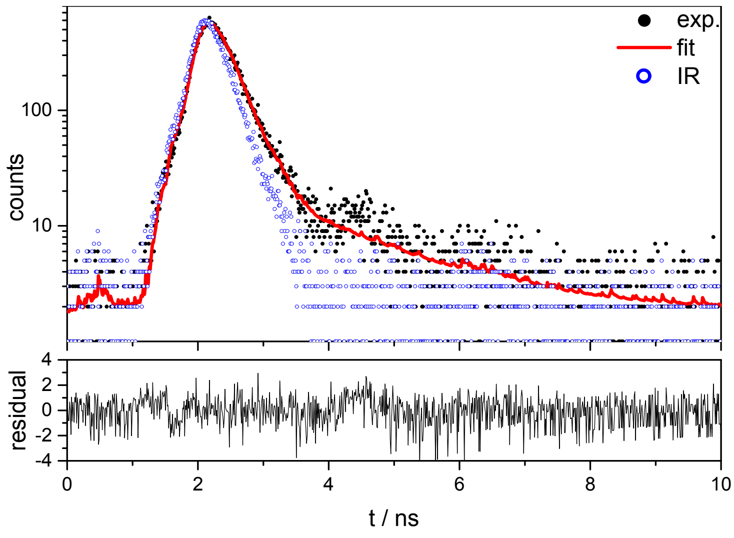

*Figure S34. Fluorescence life-time decay of **cLaP1** in aqueous suspension; instrument response (IR) shown in blue circles, experimental data in black dots and fitting function as a red line.*

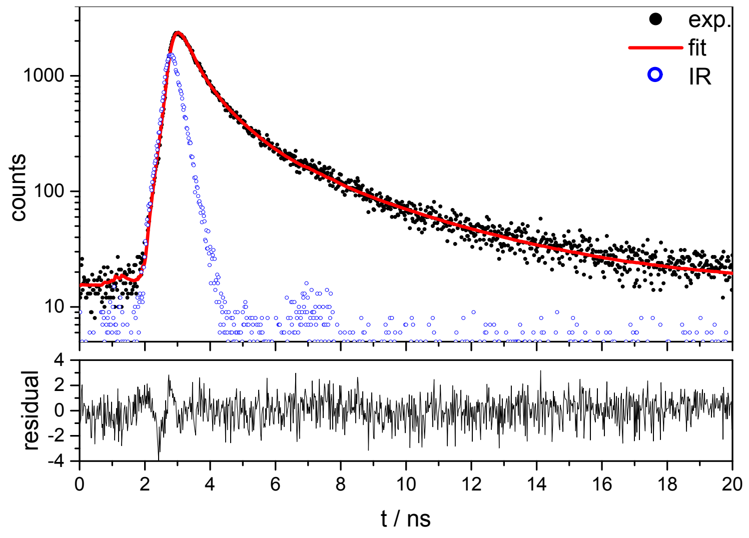

*Figure S35. Fluorescence life-time decay of **cLaP2** in aqueous suspension; instrument response (IR) shown in blue circles, experimental data in black dots and fitting function as a red line.*

Table S3.Overview of fitted and averaged fluorescence life-times in aqueous and H_2O/TEA/MeOH suspensions.

	solvent	λ_{em} / nm	τ_1 / ns	B1/ %	τ_2 / ns	B2/ %	τ_3 / ns	B3/ %	χ^2	τ_{avg} / ns
AV-6	H_2O	400	0.06	33.83	0.06	44.26	0.41	21.91	1.310	0.14
AV-7	H_2O	500	0.03	61.70	0.18	29.82	1.63	8.48	1.33	0.21
AV-20	H_2O	540	0.18	29.30	1.00	37.69	3.87	33.00	1.20	1.71

12 Transient Absorption Spectroscopy

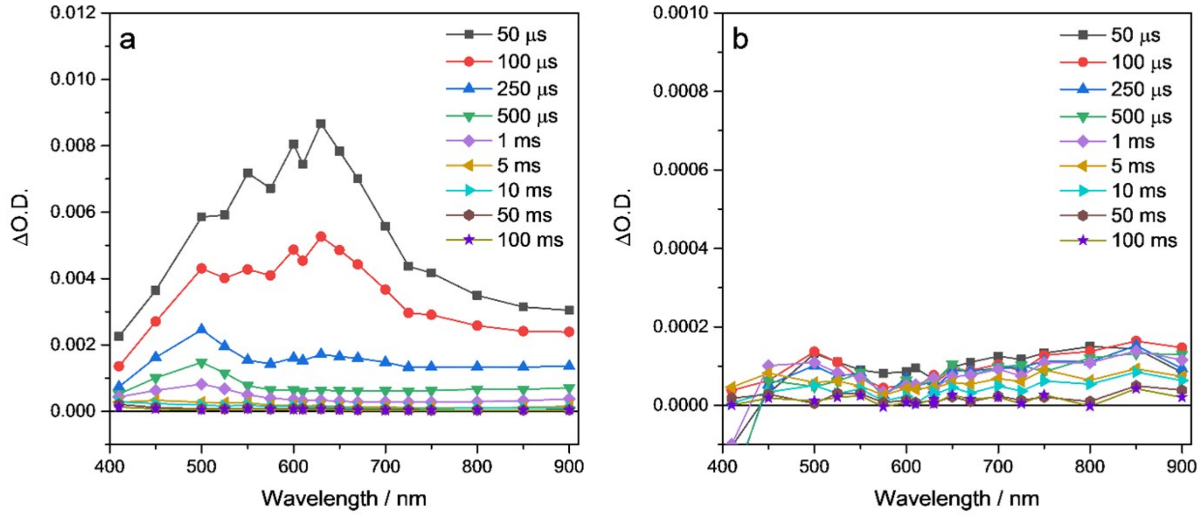

*Figure S36. μs-ms TA spectra of **cLaP1** suspended in a) H2O/TEA/MeOH and in b) H_2O which shows that the number of long-lived charges is greatly reduced in the absence of the mix system. TA spectra were recorded following excitation with a 355 nm (6 ns pulse) laser, under N_2.*

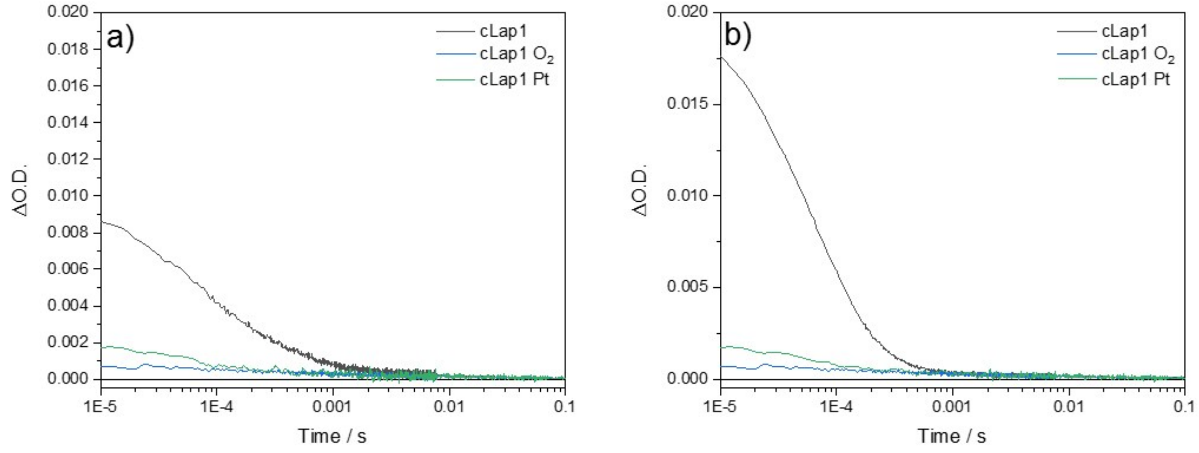

*Figure S37. μs-ms kinetic traces of **cLaP1** suspended in H2O/TEA/MeOH at probe wavelengths of a) 500 nm and b) 630 nm. TA spectra were recorded following excitation with a 355 nm laser (6 ns, 400 μJ cm^{-2}, 0.33 Hz), in N_2 (black), O_2 (blue) and with Pt in N_2 (green).*

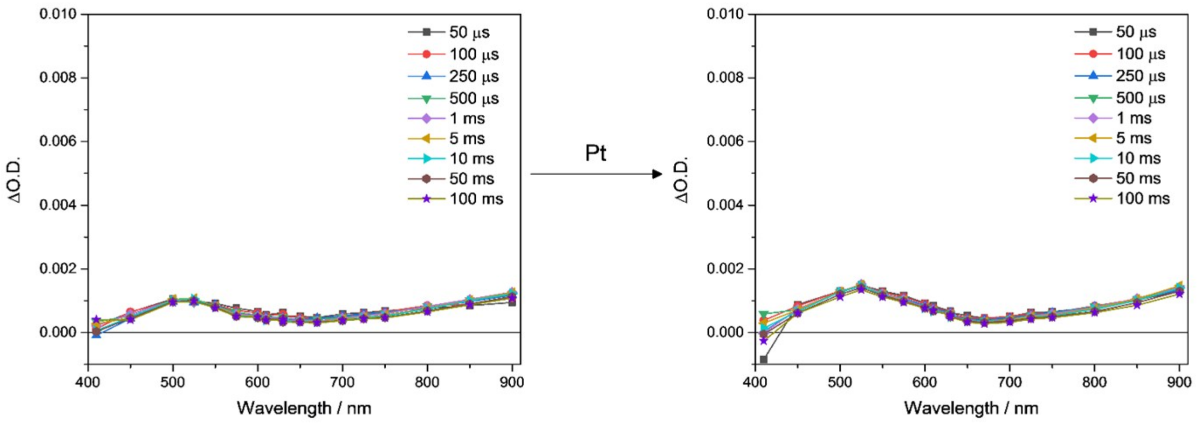

*Figure S 38. μs-ms TA spectra of **cLaP2** suspended in H_2O/TEA/MeOH before and after the addition of a Pt co-catalyst. TA spectra were recorded following excitation with a 355 nm laser (6 ns, 400 μJ cm^{-2}, 0.33 Hz), under N_2.*

13 (GFN/IPEA/sTDA)-xTB Calculations

All calculations were performed using the semi-empirical density functional tight-binding approach, xTB,[8] recently developed for the rapid calculation of geometries and optoelectronic properties of large molecular systems. The structural optimisation method (GFN-xTB[8]) is used to obtain optimized geometries from which ionisation potentials, electron affinities (obtained using IPEA-xTB[9]) and excitation energies (obtained with sTDA-xTB[10]) may be calculated. This latter method uses energy eigenvalues and wave functions obtained via xTB to calculate excited state properties. We have previously shown[11] that this semi-empirical approach, when a simple linear calibration procedure is performed using previously-obtained parameters,[11] produces absolute values of IP, EA and optical gap in excellent agreement with density functional theory.

For each polymer species (**cLaP1**, **cLaP2** & **cLiP1**), we calculate IP, EA and optical gaps for varying lengths of oligomer chains (defined by the number of aromatic rings along the polymer backbone). This is done to ensure that converged values are obtained across both ladder and non-ladder polymer species.

Table S4. Calculated optical gaps, Δ_o (lowest excitation of non-zero oscillator strength) for oligomer chains of length = 9, where 'length' is equal to the number of benzene bi-sulfoxide, thiophene or thiophene dioxide units, respectively).

Polymer	Δ_o (eV)	Oscillator Strength (a.u.)
**cLiP1**	3.625	1.379
**cLaP1**	3.061	4.544
**cLaP2**	2.880	6.057

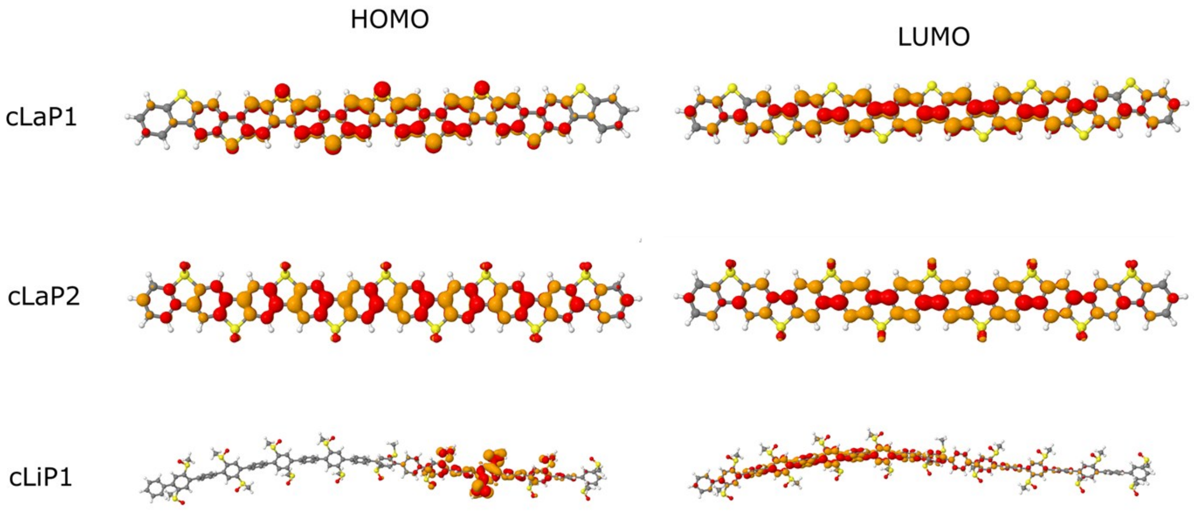

*Figure S39. Calculated HOMO and LUMO orbitals (isosurface value= 0.02) for **cLiP1**, **cLaP1** & **cLaP2**.*

14 References

1 P. Gao, X. Feng, X. Yang, V. Enkelmann, M. Baumgarten and K. Müllen, *J. Org. Chem.*, 2008, **73**, 9207–9213.

2 A. Haryono, K. Miyatake, J. Natori and E. Tsuchida, *Macromolecules*, 1999, **32**, 3146–3149.

3 a) K. Kawabata, M. Takeguchi and H. Goto, *Macromolecules*, 2013, **46**, 2078–2091; b) M. Maisuradze, G. Phalavadishvili, N. Gakhokidze, M. Matnadze, S. Tskhvitaia and E. Kalandia, *Int. J. Org. Chem.*, 2017, **07**, 34–41; c) H. Gilman and D. L. Esmay, *J. Am. Chem. Soc.*, 1952, **74**, 2021–2024;

4 T. Ishiyama, M. Murata and N. Miyaura, *J. Org. Chem.*, 1995, **60**, 7508–7510.

5 R. S. Sprick, B. Bonillo, R. Clowes, P. Guiglion, N. J. Brownbill, B. J. Slater, F. Blanc, M. A. Zwijnenburg, D. J. Adams and A. I. Cooper, *Angew. Chem. Int. Ed.*, 2016, **55**, 1792–1796.

6 J. Tauc, *Mater. Res. Bull.*, 1968, **3**, 37–46.

7 L. Wilbraham, *Code for Preparation of Tauc plots,* available at: https://github.com/ZwijnenburgGroup/taucauto.

8 S. Grimme, C. Bannwarth and P. Shushkov, *J. Chem. Theory Comput.*, 2017, **13**, 1989–2009.

9 V. Ásgeirsson, C. A. Bauer and S. Grimme, *Chem. Sci.*, 2017, **8**, 4879–4895.

10 S. Grimme and C. Bannwarth, *J. Chem. Phys.*, 2016, **145**, 54103.

11 L. Wilbraham, E. Berardo, L. Turcani, K. E. Jelfs and M. A. Zwijnenburg, *J. Chem. Inf. Model.*, 2018, Article ASAP, DOI: 10.1021/acs.jcim.8b00256.
